# Multiresolution GPC-Structured Control of a Single-Loop Cold-Flow Chemical Looping Testbed

**DOI:** 10.3390/en13071759

**Published:** 2020-04-07

**Authors:** Shu Zhang, Joseph Bentsman, Xinsheng Lou, Carl Neuschaefer, Yongseok Lee, Hamza El-Kebir

**Affiliations:** 1 Department. of Mechanical Science and Engineering, University of Illinois at Urbana-Champaign, 1206 W Green St., Urbana, IL 61801, USA; 2 Alstom Thermal Power, Windsor, CT 06095, USA; 3 Department of Aerospace Engineering, University of Illinois at Urbana-Champaign, 104 S Wright St., Urbana, IL 61801, USA

**Keywords:** chemical looping, wavelets, NARMA model, generalized predictive control (GPC)

## Abstract

Chemical looping is a near-zero emission process for generating power from coal. It is based on a multi-phase gas-solid flow and has extremely challenging nonlinear, multi-scale dynamics with jumps, producing large dynamic model uncertainty, which renders traditional robust control techniques, such as linear parameter varying *H*_∞_ design, largely inapplicable. This process complexity is addressed in the present work through the temporal and the spatiotemporal multiresolution modeling along with the corresponding model-based control laws. Namely, the nonlinear autoregressive with exogenous input model structure, nonlinear in the wavelet basis, but linear in parameters, is used to identify the dominant temporal chemical looping process dynamics. The control inputs and the wavelet model parameters are calculated by optimizing a quadratic cost function using a gradient descent method. The respective identification and tracking error convergence of the proposed self-tuning identification and control schemes, the latter using the unconstrained generalized predictive control structure, is separately ascertained through the Lyapunov stability theorem. The rate constraint on the control signal in the temporal control law is then imposed and the control topology is augmented by an additional control loop with self-tuning deadbeat controller which uses the spatiotemporal wavelet riser dynamics representation. The novelty of this work is three-fold: (1) developing the self-tuning controller design methodology that consists in embedding the real-time tunable temporal highly nonlinear, but linearly parametrizable, multiresolution system representations into the classical rate-constrained generalized predictive quadratic optimal control structure, (2) augmenting the temporal multiresolution loop by a more complex spatiotemporal multiresolution self-tuning deadbeat control loop, and (3) demonstrating the effectiveness of the proposed methodology in producing fast recursive real-time algorithms for controlling highly uncertain nonlinear multiscale processes. The latter is shown through the data from the implemented temporal and augmented spatiotemporal solutions of a difficult chemical looping cold flow tracking control problem.

## 1. Introduction

The current transition to clean power generation involves both the use of renewables, such as hydrokinetics [1], and cleaner coal-based techniques. The latter are projected to still supply power for the foreseeable future due to the abundance of coal in many industrialized and developing countries; however, they will be required to meet the hard caps on carbon emissions. Chemical looping (CL) is a near-zero emission coal-based technology in which multiple interacting loops of flowing, reactive, gas/solid mixtures produce energy via solid-oxygen carrier based combustion [2–4]. Chemical looping has remained an area of active research focused on improving its economic viability and reducing environmental footprint [5,6]. To reduce waste stream volumes and required energy, advanced optimizing control systems for the chemical looping process are required. However, the process exhibits extremely challenging nonlinear multi-scale dynamics that are hard to characterize and depend on a particular design. These features render traditional robust control techniques marginally successful in experimental trials.

The goal of the present paper is to present the development of the real-time computational control-oriented models and the corresponding model-based control design strategies found to provide the desired chemical looping tracking performance. In particular, we demonstrate a novel model-based process control methodology to control the pressure drop in the riser of a single loop chemical looping process, where the air flow rate is the controlled variable. This control approach was implemented and successfully tested on an industrial single loop cold gas/solid flow chemical looping testbed, where the previously available techniques had exhibited difficulties.

Prior to being able to control the process, it is imperative to characterize the system’s response to control inputs. Classically, this would be done by devising a physical model of the system from first principles, but this often yields limited practical utility for increasingly complex nonlinear models when viewed from the perspective of process control design. To meet this challenge, an alternative technique, identification of a model constructed on the basis of the wavelet multiresolution analysis (MRA), is used in the present work. MRA has become one of the major tools in neural networks [7–10] and nonlinear system modeling [11–18]. Wavelet-based multiresolution decomposition has been proven to constitute a universal approximator for a wide range of function spaces in terms of linear combination of scaling and wavelet functions. Wavelet approximation has no smoothness requirement on the target function, making it an appropriate candidate for identification of complex nonlinear systems with multiscale dynamics, such as those encountered in chemical looping processes. Several controller designs incorporating wavelet system representations have been proposed in the literature. Reference [18] proposed adaptive adjustment of the model resolution and the corresponding structure of the nonlinear adaptive controller. However, no optimality in controller synthesis was introduced and no testing was done on the real multiresolution system. In [19] an optimal model predictive multiresolution control law with constraints was derived. However, the controller was given as a sequence of computational steps with no clear analytical formula for controller implementation and therefore no guarantee of the acceptable real-time performance; also no adaptation was included. In [20] utilization of wavelets in generalized predictive control (GPC)l has been proposed for reduction of the computational demands on the constrained GPC, but the application was not addressing multiresolution nonlinear system modeling and was restricted to linear systems only. Thus, there has been a clearly identifiable gap in producing an optimal adaptive control law with rate constraints and guaranteed real-time performance for systems with nonlinear multiscale dynamics.

The present work fills this gap through the development of the self-tuning wavelet MRA-based topology that combines temporal and spatiotemporal loops into a single closed loop control system. The GPC structure is employed for embedding into it the identified temporal nonlinear multiscale model due to its real-timable recursive receding horizon calculation, local optimality by the virtue of being a variant of LQG [21], relatively easy incorporation of rate constraints on the control signal, tunable robustness properties (not pursued in this paper), and natural embedding of the integrator to address setpoint step changes in the chemical looping based power generation.

The paper completes the brief presentation of the results of chemical looping project at Alstom Thermal Power given in a conference publication [22] through the addition of the temporal controller derivation, and presents the previously unreleased experimental data along with the corresponding discussion, as well as the derivation and implementation of an additional spatiotemporal controller for the fast process dynamics related to the riser. The material presented is based in part on two unpublished documents, an internal technical report [23] and the PhD thesis [24] of the first author; however, the detailed controller derivation was not given in either and is presented here for the first time.

Embedding highly nonlinear, but linear in parameters, adaptable multiresolution model into GPC is a novel idea requiring nontrivial analytical effort presented in this paper. Several researchers have successfully applied GPC proposed by Clark et al. [25,26] to many control areas [27–31]. GPC, however, has limitations, some of which have been discussed by Grimble in [32]. Since GPC uses a linear dynamic model to make predictions of process outputs over the prediction horizon, its performance will significantly degrade when the real process has severe nonlinearities and runs in a wide range of operating conditions, as is the case for a chemical looping process. Therefore, it is imperative to incorporate a high fidelity nonlinear dynamic model into the GPC scheme. Accordingly, we embed a wavelet MRA model of the nonlinear single loop cold flow of the chemical looping process into the GPC scheme. Specifically, first, a single-input-single-output (SISO) nonlinear autoregressive exogenous model (NARX) based on wavelet MRA is identified on-line using the chemical looping process test rig. Then, a GPC-type performance index is formulated, which incorporates the MRA model, and a gradient descent (GD) algorithm is developed for tuning both the weighting parameters of the wavelet MRA model and the control sequence in the GPC scheme. Further, the Lyapunov function-based theorems are proven to separately guarantee the convergence of the wavelet MRA identified model and the stability of the proposed GPC scheme without constraint and provide a guidance on both controller and identifier performance tuning. A rate constraint is then imposed on the control signal to smooth out the CL process transients. The resulting controller is shown to yield a satisfactory closed loop performance over a broad operating range, effectively meeting the challenge of handling the chemical looping process complexity.

The resulting cold flow testbed control system was then further improved by augmenting the temporal closed loop structure described above with the additional spatiotemporal control of fast dynamics of the riser loop, which were not captured in the original low-frequency wavelet MRA system model. The response time of the 2-partial differential equations (2-PDE) riser model used for this purpose is much shorter than that of the identified NARX model, for which the sampling time is 1 s. Therefore, the control-oriented riser representation was obtained through the use of the 2-PDE riser model as follows: The model was simulated to obtain the riser impulse response, the latter was employed to approximate the faster dynamics of the system, and the result was used in a convolution to obtain a model of the transients. To simplify the calculations, the impulse response was approximated using Gaussian spatial and temporal wavelets. Simulation and experimental results verified the validity of the spatiotemporal wavelet-based control system topology augmentation.

The paper is organized as follow: Section 2.1 provides the nomenclature for the main variables and symbols used in the paper. Section 2.2 introduces the chemical looping process model. A NARX model representation and a wavelet MRA representation are given in Section 2.3. Section 2.4 provides derivation of a wavelet MRA-based GPC strategy for solving the tracking problem for a single loop cold flow system. The convergence of the prediction error of the wavelet MRA model identification algorithm and the tracking error of the proposed GPC control strategy are separately proven in Section 2.5. An input-constrained GPC scheme is presented in Section 2.6. Experimental results are discussed in Section 3. The closed loop topology augmentation with the spatiotemporal model-based control to account for the pressure drop DP47 over the riser related to the fluidizing air flows is presented in Section 4. The discussion of the results is presented in Section 5. A conclusion is provided in Section 6.

## 2. Materials and Methods

### 2.1. Nomenclature

*ϕ*_*j*,*k*_(*x*): orthonormal basis for *V*_*j*_; *ψ*_*j*,*k*_(*x*): orthonormal basis for *W*_*j*_; *y*(*t*): system output; *u*(*t*): system input; *w*(*t*): system noise; *e*(*t*): model output estimation error; *n*_*y*_: maximum lag in the output; *n*_*u*_: maximum lag in the input; *θ*: weighting parameter vector trained on-line; *g*_*i*_: multivariable scaling or wavelet basis function of past inputs and outputs; *γ*_*θ*_: adaptation gain for the control input vector; *U*: control input vector; Δ*u*_target_: unconstraint control signal calculated by the predictive control law; *μ*: design parameter; *ε*: voidage; *u*_*s*_: solid velocity; *U*_*g*_: superficial gas velocity; *S*_1,*w*_(*t*): control command calculated by wavelet adaptive GPC control; *y*_*r*_(*t*): reference signal; ∇_*θ*_*J*_1_(*n*): gradient of loss function *J*_1_ with respect to *θ*; ∇_*U*_*J*_2_(*n*): gradient of the loss function *J*_2_ with respect to *U*; ∇_*θ*_*e*(*n*): sensitivity derivative at time *n*.

### 2.2. Chemical Looping Process

**Figure 1.**
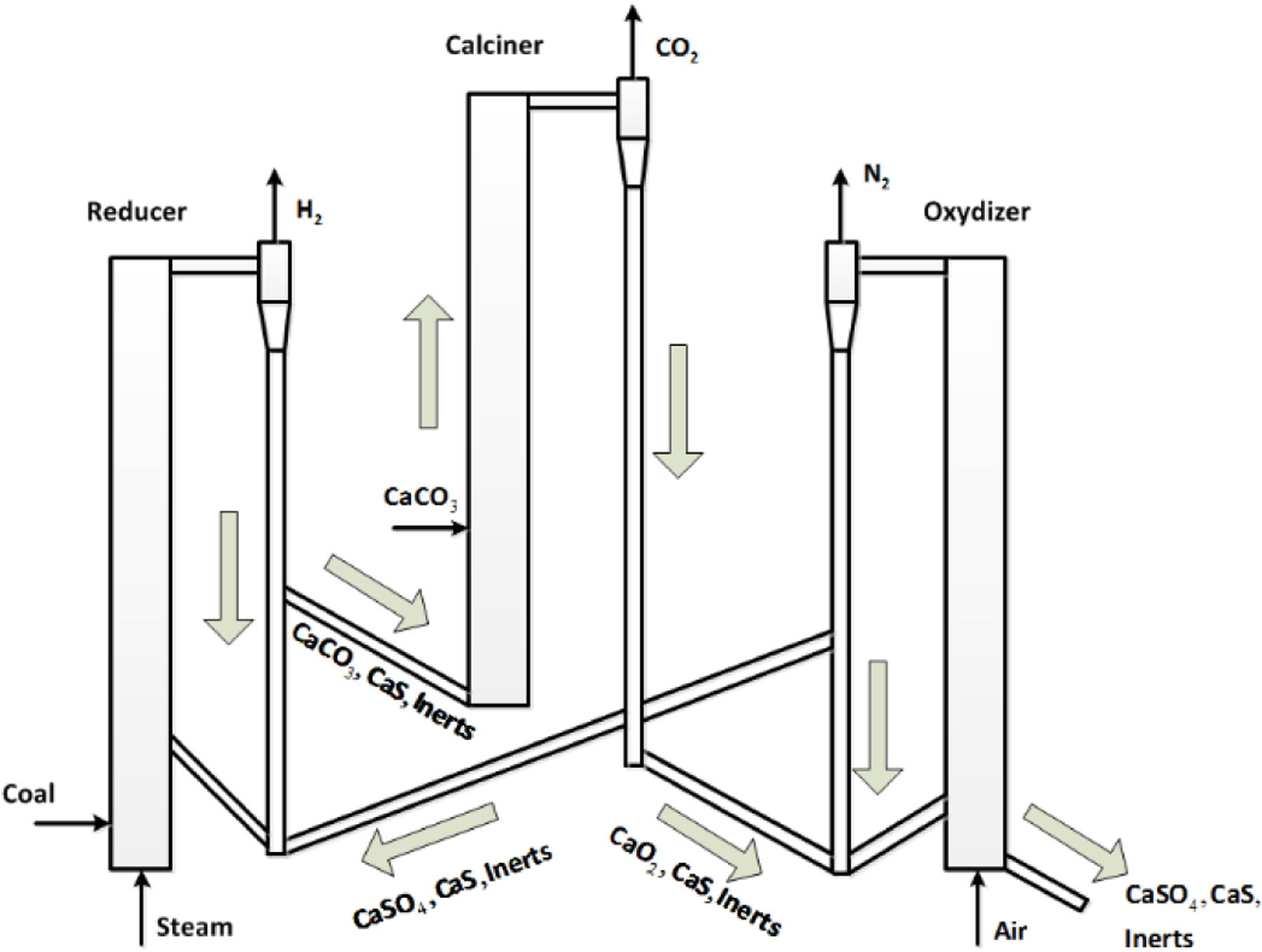
Alstom’s combustion-gasification process.

The modeling and control methodologies proposed in this paper focus on the hybrid combustion-gasification chemical looping (CL) process initially developed by Alstom Power. Chemical looping is a two-step process which first separates oxygen (O_2_) from nitrogen (N_2_) in an air stream in an air reactor. The O_2_ is transferred to a solid oxygen carrier. Next, the oxygen is carried by the solid oxide and is then used to gasify or combust solid fuel in a separate fuel reactor. As shown in Figure 1, a metal or calcium material (oxygen carrier) is burned in air forming a hot oxide (MeO_x_ or CaO_x_) in the air reactor (oxidizer). The oxygen in the hot metal oxide is used to gasify coal in the fuel reactor (reducer), thereby reducing the oxide for continuous reuse in the chemical looping cycle. CL coal power technology is an entirely new, ultra clean, low cost, high efficiency coal power plant technology for the future power market. The concept promises to be the technological link from today’s steam cycle power plants to tomorrow’s clean coal power plants, capable of high efficiency and CO_2_ capture.

The CL process with its multi-phase flows and complicated chemical reactions is characterized by process nonlinearities and time delays due to mass transport and chemical reactions. The specific operational characteristics are new and are still being studied. Hence, there is a need for further investigation and the potential for advanced control solutions. In this paper, we have focused on developing a control-oriented model for a single loop cold gas/solid flow test rig which omits all chemical reactions and interactions with other loops.

**Figure 2.**
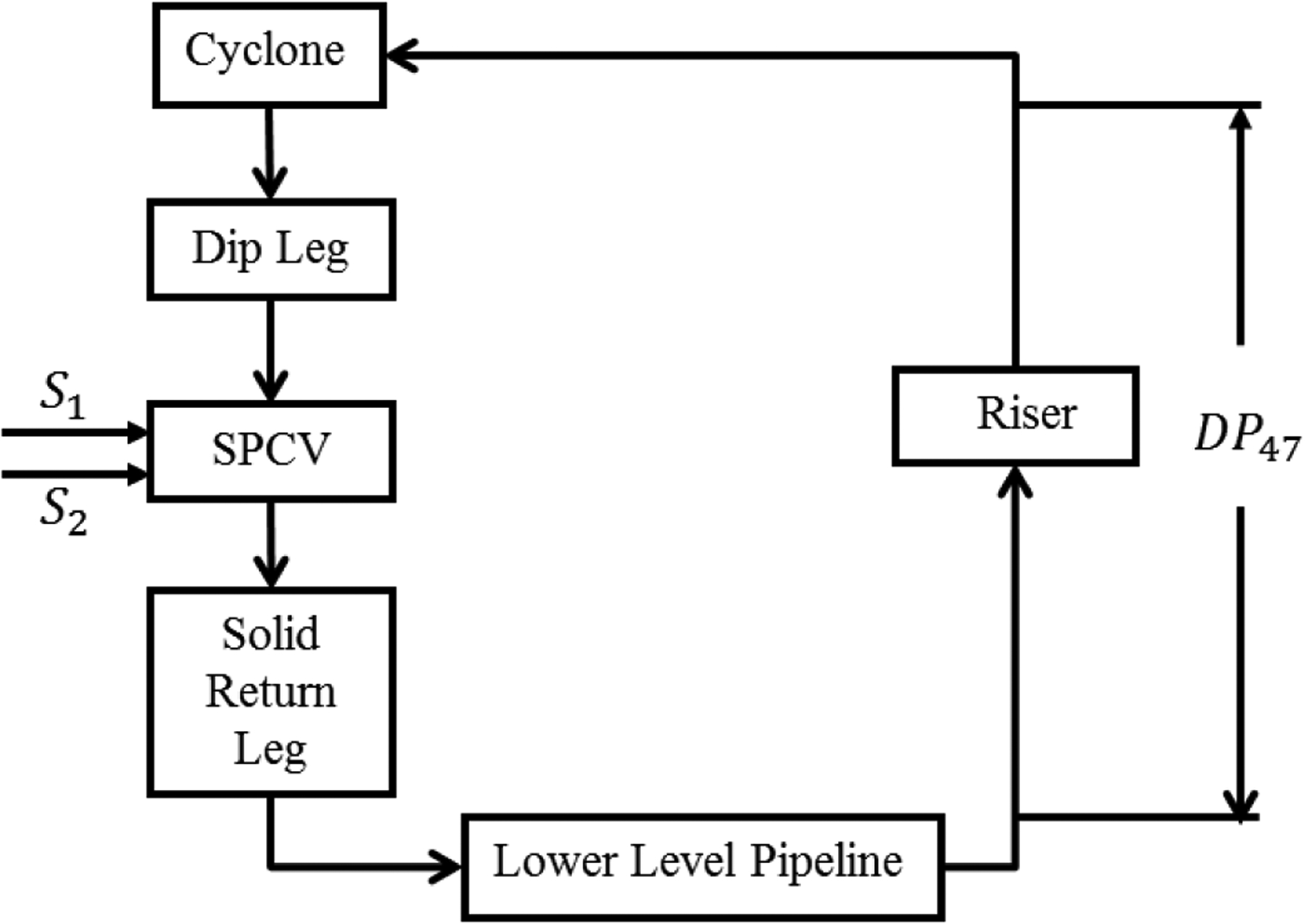
Block diagram for a single-loop cold flow CL test rig.

The block diagram of a single loop cold flow CL process is shown in Figure 2. It consists of a lower level pipeline, a riser pipeline, an upper level horizontal pipeline, a cyclone, a dip leg, seal pot control valves (SPCV), and a solid return leg. The lower level pipeline accepts air flow and solids returned from both seal pot control valves and solids which are added manually. In the riser the air-solid mixture (two-phase) flows upwards, turns into the horizontal pipeline, and then enters the cyclone. The cyclone separates the solid particles from the air. The separated solids then drop into the dip leg and enter the SPCV. The SPCV splits the solids between the return leg in its own loop and the return leg in another loop. The SPCV also maintains a pressure control boundary.

In our test rig, the manipulated variables (MV) include *S*1, *S*2—two fluidizing air flow rates into the SPCV, which change pressures in the SPCV and the flow conditions upstream and downstream of the SPCV. The controlled variable (CV) of interest is DP47, which stands for the pressure drop measured across the riser—a substantive indicator of solid/gas flow transport stability along the whole loop. The performance of the test rig implementing the controller to track a reference command was evaluated both under step-changes and cycling operation.

A wavelet MRA modelling and its embedding into a GPC-based predictive controller are described in the next two subsections.

### 2.3. Wavelet MRA Model Structure

Wavelet multiresolution analysis [14] is a function approximation tool representing function details at different scales of resolution in both the time and the frequency domains in terms of shifted and dilated scaling and wavelet functions. In general, MRA consists of a sequence of successive approximation closed subspaces *V*_*j*_ ∈ *L*_2_(*R*), *j* ∈ *Z* satisfying:

(1) $$\cdots V_{-1}\;\subset \;V_{0}\;\subset \;V_{1}\cdots ,$$

with the following properties:

(2) $$\cup_{j\in Z}V_{j}\;\text{is}\;\text{dense}\;\text{in}\;L_{2}\left(R\right);\;\cap_{j\in Z}V_{j}=\left\{0\right\},$$

(3) $$f\left(x\right)\;\in \;V_{j}\;\Leftrightarrow \;f\left(2x\right)\;\in \;V_{j+1}$$

(4) $$f\left(x\right)\;\in \;V_{j}\;\Leftrightarrow \;f\left(x-2^{-j}k\right)\;\in \;V_{j},k\;\in \;Z,$$

(5) $$V_{j}\;=\;\text{span}\left\{\phi_{j,k},k\;\in \;Z\right\},$$

where *Z* is the set of all integers, *ϕ*_*j*,*k*_(*x*) = 2^*j*/2^*ϕ*(2^*j*^*x* − *k*) is an orthonormal basis for *V*_*j*_ and *L*_2_(*R*) is the space of square integrable functions of scalar real variable.

If we define *W*_*j*_ to be the orthogonal complement of *V*_*j*_ in *V*_*j*+1_, then:

(6) $$V_{j+1}\;=\;V_{j}\;⊕\;W_{j},\;V_{j}⊥W_{j},$$

(7) $$W_{j}\;=\;\text{span}\left\{\psi_{j,k},\;k\;\in \;Z\right\},$$

where *ψ*_*j*,*k*_(*x*) = 2^*j*/2^*ψ*(2^*j*^*x* − *k*) is an orthonormal basis for *W*_*j*_. It follows from Equations (1) and (6) that, any *V*_*j*_ can be written for any *l* < *j* as:

(8) $$V_{j+1}\;=\;V_{l}\;⊕\;W_{l}\;⊕\;W_{l+1}\;⊕\;W_{l+2}\;⊕\;\cdots \;⊕\;W_{j},$$

where all the subspaces are orthogonal. Then this implies that:

(9) $$L_{2}\left(R\right)\;=\;{⊕}_{j\in Z}W_{j}$$

The functions *ϕ*_*j*,*k*_ and *ψ*_*j*,*k*_ will be referred to as scaling and wavelet functions respectively. According to Equations (8) and (9), any *f*(*x*) ∈ *L*_2_(*R*) can be represented as:

(10) f(x)=∑(f,ϕJ,n)nϕJ,n+∑(f,ψj,n)j≥J,nψj,n

The approximation starts from some lower resolution level *J* and can be truncated at certain higher resolution level *N* when:

(11) $$\left\|f\left(x\right)\;-\;\left[\underset{n}{\sum }\left(f,\phi_{J,n}\right)\phi_{J,n}\left(x\right)\;+\;\overset{N}{\underset{j=J}{\sum }}\underset{n}{\sum }\left(f,\psi_{j,n}\right)\psi_{j,n}\left(x\right)\right]\right\|<\;\varepsilon ,$$

for any predefined small error *ε* > 0.

Multivariable wavelet bases can be constructed from the tensor product of a radial basis function of a one-dimensional wavelet as described for images in [33]. Because wavelet MRA can approximate any finite energy nonlinear function to any desired accuracy level, in this paper, the wavelet MRA will be used to build the nonlinear empirical model for a single loop cold flow CL process, as shown in the next subsection.

#### The NARX Model Structure

Many systems in a variety of applications contain nonlinearities which render linear model incapable of capturing the complex dynamic system behavior. Therefore, it is of interest to develop for these applications sufficiently accurate nonlinear dynamical models. An NARX model [34] is a well-established input/output representation for nonlinear system identification. Under some mild assumptions, a discrete-time stochastic nonlinear SISO system can be expressed as:

(12) $$y\left(t\right)\;=\;f\left(y\left(t\;-\;1\right),\;\cdots \;,\;y\left(t-n_{y}\right),\;u\left(t\;-\;1\right),\;\cdots \;,\;u\left(t-n_{u}\right)\right)\;+\;w\left(t\right),$$

where *y*(*t*), *u*(*t*), *w*(*t*) are the system output, input, and noise, and *t* is discrete time, respectively, *n*_*y*_ and *n*_*u*_ are the maximum lags in the output and input, *w*(*t*) is assumed to be a zero mean, independent, and bounded noise variable, and *f*(·) is some nonlinear function. Unless some prior knowledge of the system dynamics is available, most methods use nonparametric regression to estimate the nonlinear function *f* from the data. In our case, *f* is implemented as a linear expansion in terms of the scaling and wavelet functions of regressors *g*_*i*_ such that

(13) $$f\;=\;\sum_{i=1}^{m}\theta_{i}g_{i}$$

minimizes a pre-specified approximation adequacy criterion, where *θ* = {*θ*_*i*_} is a parameter vector trained on-line, *g*_*i*_ ∈ {*ϕ*_*j*,*k*_, *ψ*_*j*,*k*_} is a multivariable scaling or wavelet basis function of past inputs and outputs, and *m* is the number of basis functions to meet some given modeling accuracy requirement.

In the next subsection, the NARX model structure introduced above is embedded into the parameter adaptation law and the GPC performance criterion, and the self-tuning MRA-based control law is derived.

### 2.4. Wavelet MRA-Based GPC Scheme

The basic methodology of GPC is to calculate the current control actions on-line at each sampling instant in order to solve a finite horizon, open-loop, optimal control problem where the first control in the optimal control sequence is applied to the plant. In this section, we present both the online wavelet MRA system identification algorithm and the GPC based predictive control strategy for the stable tracking problem of a single loop CL system. To clearly illustrate the idea of the proposed control scheme, we derive the algorithm for a SISO nonlinear dynamic system. The extension to a multi-input-multi-output setting is straightforward.

Referring to Figure 2 and its description, let DP47 be the actual system output *y* and *S*1 be the control input *u*, while *S*2 is set to a constant value. Let $\hat{y}$ denote the approximated system output. Then, the identified wavelet MRA based model is defined as follows:

(14) $$\hat{y}\left(t\right)\;=\;f\left(y\left(t\;-\;1\right),\;\cdots \;,\;y\left(t-n_{y}\right),\;u\left(t\;-\;1\right),\;\cdots \;,\;u\left(t-n_{u}\right)\right),$$

where *f* is defined in Equation (13). Then, the error between the real plant output *y* and the estimated output $\hat{y}$ is defined as:

(15) $$e\left(n\right)\;=\;y\left(n\right)\;-\;\hat{y}\left(n\right).$$

The weighting parameters *θ* in Equation (13) are trained online to minimize the loss function defined as:

(16) $$J_{1}\left(n\right)\;=\;\frac{1}{2}e^{2}\left(n\right),$$

where *n* indicates discrete time. To make *J*_1_ small, we employ a parameter adaptation law in the form of a gradient descent (GD) algorithm, which adjusts the weighting gains *θ* to keep the gradient of *J*_1_ negative, that is:

(17) $$\theta \left(n\;+\;1\right)\;=\;\theta \left(n\right)\;-\;\gamma_{\theta }\nabla_{\theta }J_{1}\left(n\right)\;=\;\theta \left(n\right)\;-\;\gamma_{\theta }e\left(n\right)\nabla_{\theta }e\left(n\right),$$

where *γ*_*θ*_ is the adaptation gain, ∇_*θ*_*J*_1_(*n*) is the gradient of *J*_1_ with respect to *θ* at discrete time *n*, and ∇_*θ*_*e*(*n*) is the so-called sensitivity derivative at time *n* indicating how the error is influenced by the weighting parameters *θ*. From Equations (13)–(15), the sensitivity derivative ∇_*θ*_*e* can be derived as follows:

(18) $$\nabla_{\theta }e\;=\;-\nabla_{\theta }\hat{y}\;=\;-\nabla_{\theta }f\;=\;-g\Rightarrow \theta \left(n\;+\;1\right)\;=\;\theta \left(n\right)\;+\;\gamma_{\theta }e\left(n\right)g\left(n\right).$$

Suppose the future set-point signals *y*_*m*_(*n* + *k*), *k* = 1, 2, ⋯ are available. In the context of GPC, define another loss function as follows:

(19) $$J_{2}\;=\;\frac{1}{2}\left\{\sum_{k=N_{1}}^{N_{2}}{\left(y_{m}\left(n\;+\;k\right)\;-\;\hat{y}\left(n\;+\;k\right)\right)}^{2}\;+\;\sum_{k=1}^{N_{u}}\rho_{k}\Delta u{\left(n+k-1\right)}^{2}\right\},$$

where *N*_1_ and *N*_2_ are the minimum and the maximum output prediction horizons, respectively, *N*_*u*_ is the control horizon, Δ is the difference operator, Δ*u*(*n*) = *u*(*n*) − *u*(*n* − 1), and *ρ*_*k*_ is the *k*-th control weighting factor. Assuming *N*_1_ = 1, *N*_2_ = *L* = *N*_*p*_, and identical control weighing factor *ρ*_*k*_ = *ρ*, Equation (19) can be rewritten in the vector form as:

(20) $$J_{2}\;=\;\frac{1}{2}\left\{{\left\|Y_{m}\left(n\;+\;1\right)\;-\;\hat{Y}\left(n\;+\;1\right)\right\|}^{2}\;+\;\rho \|\Delta U\left(n\right){\|}^{2}\right\},$$

where:

$$Y_{m}\left(n\;+\;1\right)\;=\;{\left[y_{m}\left(n\;+\;1\right),\;y_{m}\left(n\;+\;2\right),\;\cdots \;,\;y_{m}\left(n\;+\;L\right)\right]}^{T},\;\hat{Y}\left(n\;+\;1\right)\;=\;{\left[\hat{y}\left(n\;+\;1\right),\;\hat{y}\left(n\;+\;2\right),\;\cdots \;,\;\hat{y}\left(n\;+\;L\right)\right]}^{T},\;U\left(n\right)\;=\;{\left[u\left(n\right),\;u\left(n\;+\;1\right),\;\cdots \;,\;u\left(n\;+\;N_{u}\;-\;1\right)\right]}^{T},\;\Delta U\left(n\right)={\left[\Delta u\left(n\right),\;\Delta u\left(n\;+\;1\right),\;\cdots \;,\;\Delta u\left(n+N_{u}-1\right)\right]}^{T},$$

and ‖ · ‖ is the *L*_2_ norm of the *n*-dimensional real vectors.

The loss function *J*_2_ is now minimized to drive the system output *y* to the reference signal *y*_*m*_ given that the wavelet MRA identifier accurately approximates the real process dynamics on-line. At each sampling instant, an optimal control sequence is calculated using future predicted output values of the identified model, but only the first one is applied to the system. To minimize *J*_2_, the GD method is implemented again to recursively calculate the *N*_*u*_-dimensional control increment sequence Δ*U* as follows:

(21) $$\Delta U\left(n\right)\;=\;-\gamma_{u}\nabla_{U}J_{2}\left(n\right),$$

where *γ*_*u*_ is the adaptation gain for the control input vector *U*. Noting that for any vector *y*(*x*), ∇_*x*_‖ *y* ‖^2^ = 2(∇_*x*_*y*)*y*, from Equations (20) and (21), the gradient of the loss function *J*_2_ with respect to *U* can be obtained as:

$$\nabla_{U}J_{2}\left(n\right)\;=\;\frac{1}{2}\nabla_{U}\left\{{\left\|Y_{m}\left(n\;+\;1\right)\;-\;\hat{Y}\left(n\;+\;1\right)\right\|}^{2}\;+\;\rho {\left\|\Delta U\left(n\right)\right\|}^{2}\right\}\;=\;\nabla_{u}\left\{Y_{m}\left(n\;+\;1\right)\;-\;\hat{Y}\left(n\;+\;1\right)\right\}\left\{Y_{m}\left(n\;+\;1\right)\;-\;\hat{Y}\left(n\;+\;1\right)\right\}\;+\;\rho \nabla_{U}\left\{\Delta U\left(n\right)\right\}\left\{\Delta U\left(n\right)\right\}.$$

The first part of the expression above is evaluated as follows. First, we note that since *y*_*m*_(*n*) are the reference signals, which are preset constants, we have ∂*y*_*m*_(*n* + 1)/∂*u*(*n*) = 0. Then, since $\hat{y}\left(n\;+\;k\right)\;=\;\theta^{T}u,\;\hat{y}$ depends only on the past *u*, we have:

$$\frac{\partial y\left(n\;+\;k\right)}{\partial u\left(n\;+\;l\right)}\;=\;\{\begin{array}{c} \frac{\partial \hat{y}\left(n\;+\;k\right)}{\partial u\left(n\;+\;l\right)},\text{ when}\;k\;>\;l,\\ 0,\text{ when}\;k\;\leq \;l. \end{array}$$

This yields:

$$\nabla_{U}\left\{Y_{m}\left(n\;+\;1\right)\;-\;\hat{Y}\left(n\;+\;1\right)\right\}\;=\;{\left[\begin{array}{cccc} \frac{\partial }{\partial u\left(n\right)}\left\{y_{m}\left(n\;+\;1\right)\;-\;\hat{y}\left(n\;+\;1\right)\right\} & \frac{\partial }{\partial u\left(n\right)}\left\{y_{m}\left(n\;+\;2\right)\;-\;\hat{y}\left(n\;+\;2\right)\right\} & \cdots & \frac{\partial }{\partial u\left(n\right)}\left\{y_{m}\left(n\;+\;L\right)\;-\;\hat{y}\left(n\;+\;L\right)\right\}\\ \frac{\partial }{\partial u\left(n\;+\;1\right)}\left\{y_{m}\left(n\;+\;1\right)\;-\;\hat{y}\left(n\;+\;1\right)\right\} & \frac{\partial }{\partial u\left(n\;+\;1\right)}\left\{y_{m}\left(n\;+\;2\right)\;-\;\hat{y}\left(n\;+\;2\right)\right\} & \cdots & \frac{\partial }{\partial u\left(n\;+\;1\right)}\left\{y_{m}\left(n\;+\;L\right)\;-\;\hat{y}\left(n\;+\;L\right)\right\}\\ \vdots & \vdots & \ddots & \vdots \\ \frac{\partial }{\partial u\left(n+N_{u}-1\right)}\left\{y_{m}\left(n\;+\;1\right)\;-\;\hat{y}\left(n\;+\;1\right)\right\} & \frac{\partial }{\partial u\left(n+N_{u}-1\right)}\left\{y_{m}\left(n\;+\;2\right)\;-\;\hat{y}\left(n\;+\;2\right)\right\} & \cdots & \frac{\partial }{\partial u\left(n+N_{u}-1\right)}\left\{y_{m}\left(n\;+\;L\right)\;-\;\hat{y}\left(n\;+\;L\right)\right\} \end{array}\right]}_{N_{u}\times N_{p}}\;=\;{\left[\begin{array}{cccccccc} -\frac{\partial \hat{y}\left(n\;+\;1\right)}{\partial u\left(n\right)} & & -\frac{\partial \hat{y}\left(n\;+\;2\right)}{\partial u\left(n\right)} & \cdots & -\frac{\partial \hat{y}\left(n+N_{u}\right)}{\partial u\left(n\right)} & & \cdots & -\frac{\partial \hat{y}\left(n\;+\;L\right)}{\partial u\left(n\right)}\\ 0 & & -\frac{\partial \hat{y}\left(n\;+\;2\right)}{\partial u\left(n\;+\;1\right)} & \cdots & -\frac{\partial \hat{y}\left(n+Nu\right)}{\partial u\left(n\;+\;1\right)} & & \cdots & -\frac{\partial \hat{y}\left(n\;+\;L\right)}{\partial u\left(n\;+\;1\right)}\\ & & & & \vdots & & & \\ 0 & \cdots & 0 & & -\frac{\partial \hat{y}\left(n+N_{u}\right)}{\partial u\left(n+N_{u}-1\right)} & \cdots & & -\frac{\partial \hat{y}\left(n\;+\;L\right)}{\partial u\left(n+N_{u}-1\right)} \end{array}\right]}_{N_{u}\times N_{p}}$$

The second part of ∇_*U*_*J*_2_(*n*) is evaluated by taking into account the relation Δ*u*(*n*) = *u*(*n*)−*u*(*n*−1), so that ∂Δ*u*(*n*)/∂*u*(*n*) = 1 and ∂Δ*u*(*n*)/∂*u*(*n* − 1) = −1. The latter yields:

(22) $$\nabla_{U}\left\{\Delta U\left(n\right)\right\}={\left[\begin{array}{ccccc} \frac{\partial \Delta u\left(n\right)}{\partial u\left(n\right)} & \frac{\partial \Delta u\left(n\;+\;1\right)}{\partial u\left(n\right)} & \frac{\partial \Delta u\left(n\;+\;2\right)}{\partial u\left(n\right)} & \cdots & \frac{\partial \Delta u\left(n+N_{u}-1\right)}{\partial u\left(n\right)}\\ \frac{\partial \Delta u\left(n\right)}{\partial u\left(n\;+\;1\right)} & \frac{\partial \Delta u\left(n\;+\;1\right)}{\partial u\left(n\;+\;1\right)} & \frac{\partial \Delta u\left(n\;+\;2\right)}{\partial u\left(n\;+\;1\right)} & \cdots & \frac{\partial \Delta u\left(n+Nu-1\right)}{\partial u\left(n\;+\;1\right)}\\ \vdots & \vdots & \vdots & \ddots & \vdots \\ \frac{\partial \Delta u\left(n\right)}{\partial u\left(n+N_{u}-1\right)} & \cdots & \frac{\partial \Delta u\left(n+N_{u}-3\right)}{\partial u\left(n+N_{u}-1\right)} & \frac{\partial \Delta u\left(n+N_{u}-2\right)}{\partial u\left(n+N_{u}-1\right)} & \frac{\partial \Delta u\left(n+N_{u}-1\right)}{\partial u\left(n+N_{u}-1\right)} \end{array}\right]}_{N_{u}\times N_{u}}\;={\left[\begin{array}{ccccc} 1 & -1 & 0 & \cdots & 0\\ 0 & 1 & -1 & \cdots & 0\\ \vdots & \vdots & \vdots & \vdots & \vdots \\ 0 & \cdots & 0 & 0 & 1 \end{array}\right]}_{N_{u}\times N_{u}}.$$

**Figure 3.**
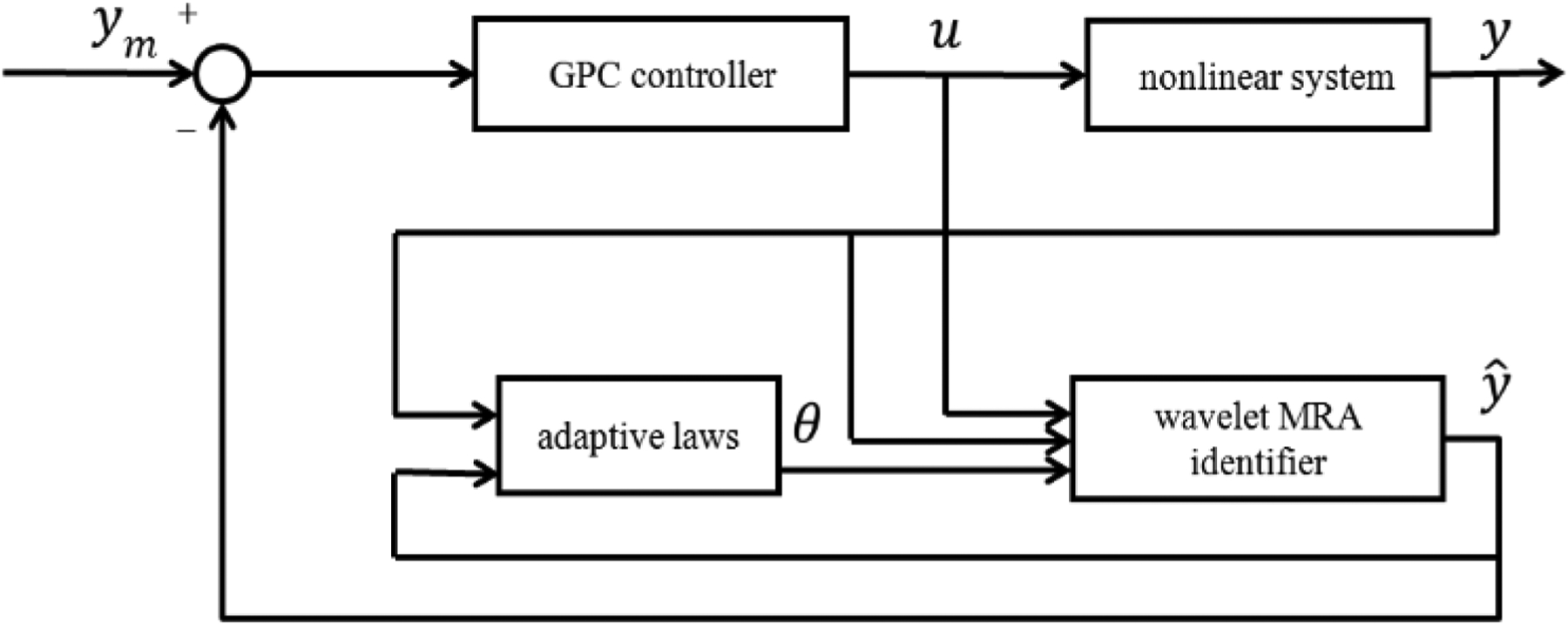
Schemetic of the wavelet MRA-based self-tuning GPC control system.

Combining the above expressions into:

$$\nabla_{U}J_{2}\left(n\right)\;=\;-G\left(Y_{m}\left(n\;+\;1\right)\;-\;\hat{Y}\left(n\;+\;1\right)\right)+\rho H\Delta U\left(n\right),$$

and substituting into Equation (21) as:

(23) $$\Delta U\left(n\right)\;=\;-\gamma_{u}\nabla_{U}J_{2}\left(n\right)\;=\;\gamma_{u}G\left(Y_{m}\left(n\;+\;1\right)\;-\;\hat{Y}\left(n\;+\;1\right)\right)-\gamma_{u}\rho H\Delta U\left(n\right),$$

where:

(24) $$G\;=\;{\left[\begin{array}{cccccccc} \frac{\partial \hat{y}\left(n\;+\;1\right)}{\partial u\left(n\right)} & & \frac{\partial \hat{y}\left(n\;+\;2\right)}{\partial u\left(n\right)} & \cdots & \frac{\partial \hat{y}\left(n+Nu\right)}{\partial u\left(n\right)} & & \cdots & \frac{\partial \hat{y}\left(n\;+\;L\right)}{\partial u\left(n\right)}\\ 0 & & \frac{\partial \hat{y}\left(n\;+\;2\right)}{\partial u\left(n\;+\;1\right)} & \cdots & \frac{\partial \hat{y}\left(n+Nu\right)}{\partial u\left(n\;+\;1\right)} & & \cdots & \frac{\partial \hat{y}\left(n\;+\;L\right)}{\partial u\left(n\;+\;1\right)}\\ & & & & \vdots & & & \\ 0 & \cdots & 0 & & \frac{\partial \hat{y}\left(n+Nu\right)}{\partial u\left(n+Nu-1\right)} & \cdots & & \frac{\partial \hat{y}\left(n\;+\;L\right)}{\partial u\left(n+Nu-1\right)} \end{array}\right]}_{Nu\times Np}$$

and:

(25) $$H\;=\;{\left[\begin{array}{ccccc} 1 & -1 & 0 & \cdots & 0\\ 0 & 1 & -1 & \cdots & 0\\ \vdots & \vdots & \vdots & \vdots & \vdots \\ 0 & \cdots & 0 & 0 & 1 \end{array}\right]}_{N_{u}\times N_{u}}$$

yields the control law of the form:

(26) $$\Delta U\left(n\right)\;=\;{\left(I\;+\;\gamma_{u}\rho H\right)}^{-1}\gamma_{u}G\left(Y_{m}\left(n\;+\;1\right)\;-\;\hat{Y}\left(n\;+\;1\right)\right),$$

where *I* is the *N*_*u*_ × *N*_*u*_ identity matrix. *G* can be computed from the chosen wavelet MRA model structure. The proposed wavelet MRA model-based GPC control schematic is shown in Figure 3. As a result, the tracking problem for a single loop cold flow system can be solved by the wavelet MRA-based GPC control strategy using the convergence tuning guidelines developed in the next section.

### 2.5. Convergence and Stability

In this section, we show the output error convergence of the wavelet MRA model identification algorithm and the tracking error convergence of the proposed GPC-based control strategy. These proofs serve to show that the system identification scheme will converge to the true system model of the preselected resolution, while the predictive control scheme will provide tracking of the desired output by the system output. The adaptive identification and control laws have one parameter each in the form of the adaptation gains chosen by the user. It has been shown [35] that adaptation gains are crucial to the stability and performance of an adaptive control system. Therefore, we have provided analytic guidelines for selecting these gains. The validity of such separate convergence analysis is certainly limited under significant coupling between identification and control (e.g., for aggressively chosen gains), and the coupled analysis will be reported elsewhere. However, the results are well supported by the actual implementation and testing.

#### 2.5.1. Convergence of Wavelet MRA Identifier

Define a discrete-type Lyapunov function as:

(27) $$V_{1}\left(n\right)\;=\;\frac{1}{2}e^{2}\left(n\right),$$

where *e*(*n*) defined in Equation (15) represents the output modeling error. Then, the increment of the Lyapunov function is given by:

(28) $$\Delta V_{1}\left(n\right)\;=\;V_{1}\left(n\;+\;1\right)\;-\;V_{1}\left(n\right)\;=\;\frac{1}{2}\left(e^{2}\left(n\;+\;1\right)\;-\;e^{2}\left(n\right)\right).$$

The error difference can be represented using the Jacobian matrix by:

(29) $$\Delta e\left(n\right)\;=\;e\left(n\;+\;1\right)\;-\;e\left(n\right)\;=\;\left[\frac{\partial e\left(n\right)}{\partial \theta \left(n\right)}\right]\Delta \theta \left(n\right)$$

where $\Delta \theta \left(n\right)\;=\;{\left\{\Delta \theta_{i}\left(n\right)\right\}}_{i=1}^{m}$ represents a change in the arbitrary component of the weighting gain vector *θ*. From Equation (18), Δ*θ*_*i*_(*n*) can be obtained by:

(30) $$\Delta \theta \left(n\right)\;=\;\gamma_{\theta }e\left(n\right)g\left(n\right),$$

(31) $$\left[\frac{\partial e\left(n\right)}{\partial \theta \left(n\right)}\right]\;=\;-\left[\frac{\partial \hat{y}\left(n\right)}{\partial \theta \left(n\right)}\right]\;=\;-g^{T}\left(n\right),$$

where $g\left(n\right)\;=\;{\left\{g_{i}\left(n\right)\right\}}_{i=1}^{m}$.

##### Theorem 1.

Let *γ_θ_* be the adaptation gain for the weights of the wavelet MRA identified model and *g*_max_ be defined as *g*_max_ ≔ max*_n_* ‖ *g*(*n*) ‖, where *g* is the wavelet MRA basis function, *n* is the discrete time index, and ‖ · ‖ is the *L*_2_ norm of a real vector. Then convergence is guaranteed if *γ_θ_* is chosen as:

(32) $$0\;<\;\gamma_{\theta }\;<\;\frac{2}{g_{\text{max}}^{2}}$$

**Proof.** From Equations (28)–(31), Δ*V*_1_(*n*) can be represented as:

(33) $$\Delta V_{1}\left(n\right)\;=\;\Delta e\left(n\right)\left[e(n)\;+\;\frac{1}{2}\Delta e\left(n\right)\right]\;=\;\left[\frac{\partial e\left(n\right)}{\partial \theta }\right]\gamma_{\theta }e(n)g\left(n\right)\;\times \;\left\{e\left(n\right)\;+\;\frac{1}{2}\left[\frac{\partial e\left(n\right)}{\partial \theta }\right]\gamma_{\theta }e\left(n\right)g\left(n\right)\right\}\;=\;-\gamma_{\theta }e^{2}\left(n\right)\|g\left(n\right){\|}^{2}+\frac{1}{2}\gamma_{\theta }^{2}e^{2}\left(n\right)\|g\left(n\right){\|}^{4}=-\lambda e^{2}\left(n\right),$$

where:

(34) $$\lambda \;=\;\frac{1}{2}\gamma_{\theta }\|g\left(n\right){\|}^{2}\left(2-\gamma_{\theta }\|g\left(n\right){\|}^{2}\right)\geq \;\frac{1}{2}\gamma_{\theta }\|g\left(n\right){\|}^{2}\left(2-\gamma_{\theta }g_{\text{max}}^{2}\right)\;>\;0.$$

From Equation (32) we obtain *V*_1_(*n*) ≥ 0 and Δ*V*_1_(*n*) < 0, then the convergence of the weighting parameters of the identified wavelet MRA model is guaranteed. □

#### 2.5.2. Stability Analysis of Wavelet MRA-Based GPC

Define a second discrete Lyapunov function as:

(35) $$V_{2}\left(n\right)\;=\;\frac{1}{2}\|E\left(n\;+\;1\right){\|}^{2},$$

where $E\left(n\;+\;1\right)\;=\;Y_{m}\left(n\;+\;1\right)\;-\;\hat{Y}\left(n\;+\;1\right)$. Then the change of the Lyapunov function is obtained as:

(36) $$\Delta V_{2}\left(n\right)=V_{2}\left(n\;+\;1\right)\;-\;V_{2}\left(n\right)=\frac{1}{2}\left(\|E\left(n\;+\;2\right){\|}^{2}\;-\;\|E\left(n\;+\;1\right){\|}^{2}\right).$$

Similarly to Equation (29), the error difference can be represented using the Jacobian matrix by:

(37) $$\Delta E\left(n\;+\;1\right)\;=\;E\left(n\;+\;2\right)\;-\;E\left(n\;+\;1\right)\;=\;\left[\frac{\partial E\left(n\;+\;1\right)}{\partial U(n)}\right]\Delta U\left(n\right),$$

where Δ*U*(*n*) is defined in Equation (26) and $\frac{\partial E\left(n\;+\;1\right)}{\partial U\left(n\right)}\;=\;-G^{T}$. Then Equation (37) can be expressed as:

(38) $$\Delta E\left(n\;+\;1\right)\;=\;-G^{T}{\left(I+\gamma_{u}\rho H\right)}^{-1}\gamma_{u}GE\left(n\;+\;1\right).$$

##### Theorem 2.

Let γ_u_ be the adaptation gain for the GPC control input sequence. Assume a control weighting factor *ρ* > 0. Then the stable tracking convergence of the wavelet MRA based GPC control system is guaranteed if:

(39) $$0\;<\;\gamma_{u}\;<\;\frac{2}{\lambda_{\text{max}}\left(GG^{T}\right)},$$

where *λ*_max_(·) is the maximum eigenvalue of the matrix.

**Proof.** From Equations (36)–(38), Δ*V*_2_(*n*) can be represented as:

(40) $$\Delta V_{2}\left(n\right)\;=\;\frac{1}{2}\left[{\left(E\left(n\;+\;1\right)\;+\;\Delta E\left(n\;+\;1\right)\right)}^{T}(E\left(n\;+\;1\right)\;+\;\Delta E\left(n\;+\;1\right))\;-\;E{\left(n\;+\;1\right)}^{T}E\left(n\;+\;1\right)\right]\;=\;\Delta E^{T}\left(n\;+\;1\right)\left[E\left(n\;+\;1\right)\;+\;\frac{1}{2}\Delta E\left(n\;+\;1\right)\right]\;=\;-{\left(GE\right)}^{T}\gamma_{u}{\left({\left(I+\gamma_{u}\rho H\right)}^{-1}\right)}^{T}\left[I-\frac{1}{2}GG^{T}{\left(I+\gamma_{u}\rho H\right)}^{-1}\gamma_{u}\right]GE\;=\;-{\left(GE\right)}^{T}R_{1}R_{2}GE,$$

where:

(41) $$R_{1}\;=\;\gamma_{u}{\left({\left(I+\gamma_{u}\rho H\right)}^{-1}\right)}^{T},$$

(42) $$R_{2}\;=\;I\;-\;\frac{1}{2}\gamma_{u}GG^{T}{\left(I\;+\;\gamma_{u}\rho H\right)}^{-1}.$$

□

If *R*_1_ and *R*_2_ are both positive definite matrices, it follows that Δ*V*_2_(*n*) < 0. Together with *V*_2_(*n*) > 0, the stable tracking of the reference signals is guaranteed.

From Equation (25) it can be shown that the eigenvalues of *H* are $\lambda_{H}\;=\;{\left\{1,\;\cdots \;,\;1\right\}}_{N_{u}\times 1}$. Then the eigenvalues of *R*_1_ can be derived as:

(43) $$\lambda_{R_{1}}\;=\;{\left\{\gamma_{u}{\left(1+\gamma_{u}\rho \right)}^{-1},\;\cdots \;,\;\gamma_{u}{\left(1\;+\;\gamma_{u}\rho \right)}^{-1}\right\}}_{N_{u}\times 1}$$

Hence, all eigenvalues of *R*_1_ are positive if *γ*_*u*_ > 0. It follows that *R*_1_ > 0.

If $0\;<\;\gamma_{u}\;<\;\frac{2}{\lambda_{\text{max}}\left(GG^{T}\right)}$, then:

(44) $$I\;-\;\frac{1}{2}\gamma_{u}GG^{T}\;>\;0.$$

From Equation (25) we have:

(45) $$\gamma_{u}\rho H\;>\;0.$$

Then from Equations (44) and (45)

(46) $$I\;-\;\frac{1}{2}\gamma_{u}GG^{T}\;+\;\gamma_{u}\rho H\;>\;0.$$

Similarly to the way it was done for Equation (43), we can prove that *I* + *γ*_*u*_*ρH* > 0. Then Equation (46) can be rewritten as:

(47) $$\left(I\;+\;\gamma_{u}\rho H\right)\left(I\;-\;\frac{1}{2}\gamma_{u}GG^{T}{\left(I\;+\;\gamma_{u}\rho H\right)}^{-1}\right)\;>\;0.$$

Since *I* + *γ*_*u*_*ρH* > 0, $\left(I\;-\;\frac{1}{2}\gamma_{u}GG^{T}{\left(I\;+\;\gamma_{u}\rho H\right)}^{-1}\right)\;>\;0$ follows. Now we have *V*_2_(*n*) ≥ 0 and Δ*V*_2_(*n*) < 0. With this, the convergence of the prediction error of the wavelet MRA model identification algorithm and the tracking error of the proposed GPC control strategy have been separately proven.

### 2.6. Wavelet MRA GPC with Input Constraints

The stability analysis in Section 2.5 does not account for constraints. In practice, all process inputs are subject to certain constraints due to the actuation limits. In [36], two types of constraints are considered in the GPC design procedure, namely the rate and the magnitude limits on the input control signal, given, respectively, by:

(48) $$\Delta u_{\text{min}}\;\leq \;u\left(n\;+\;k\right)\;-\;u\left(n\;+\;k\;-\;1\right)\;\leq \;\Delta u_{\text{max}},$$

(49) $$u_{\text{min}}\;\leq \;u\left(n\;+\;k\right)\;\leq \;u_{\text{max}},$$

where 0 ≤ *k* ≤ *N*_*u*_ − 1. When constraints are included, the stability properties obtained above must be reanalyzed. The stability analysis for constrained wavelet MRA–GPC architecture will be addressed elsewhere. Taking into account the CL process actuator constraints, the control input *u* is subject to an input magnitude constraint saturation:

(50) $$u^{*}\left(n\right)\;=\;\text{sat}\left[u\left(n\right)\right]\;=\;\{\begin{array}{c} u_{\text{min}}\text{ if}\;u\left(n\right)\;<\;u_{\text{min}}\\ u_{\text{max}}\text{ if}\;u\left(n\right)\;>\;u_{\text{max}}\\ u\left(n\right)\text{ otherwise } \end{array}.$$

In the experiments, the latter constraints were seldomly attained, whereas control rate constraints of the form of Equation (48) had to be introduced to achieve good experimental results, as presented in the next section.

## 3. Results

### 3.1. MRA Temporal Modeling of the Chemical Looping Process Testbed

**Figure 4.**
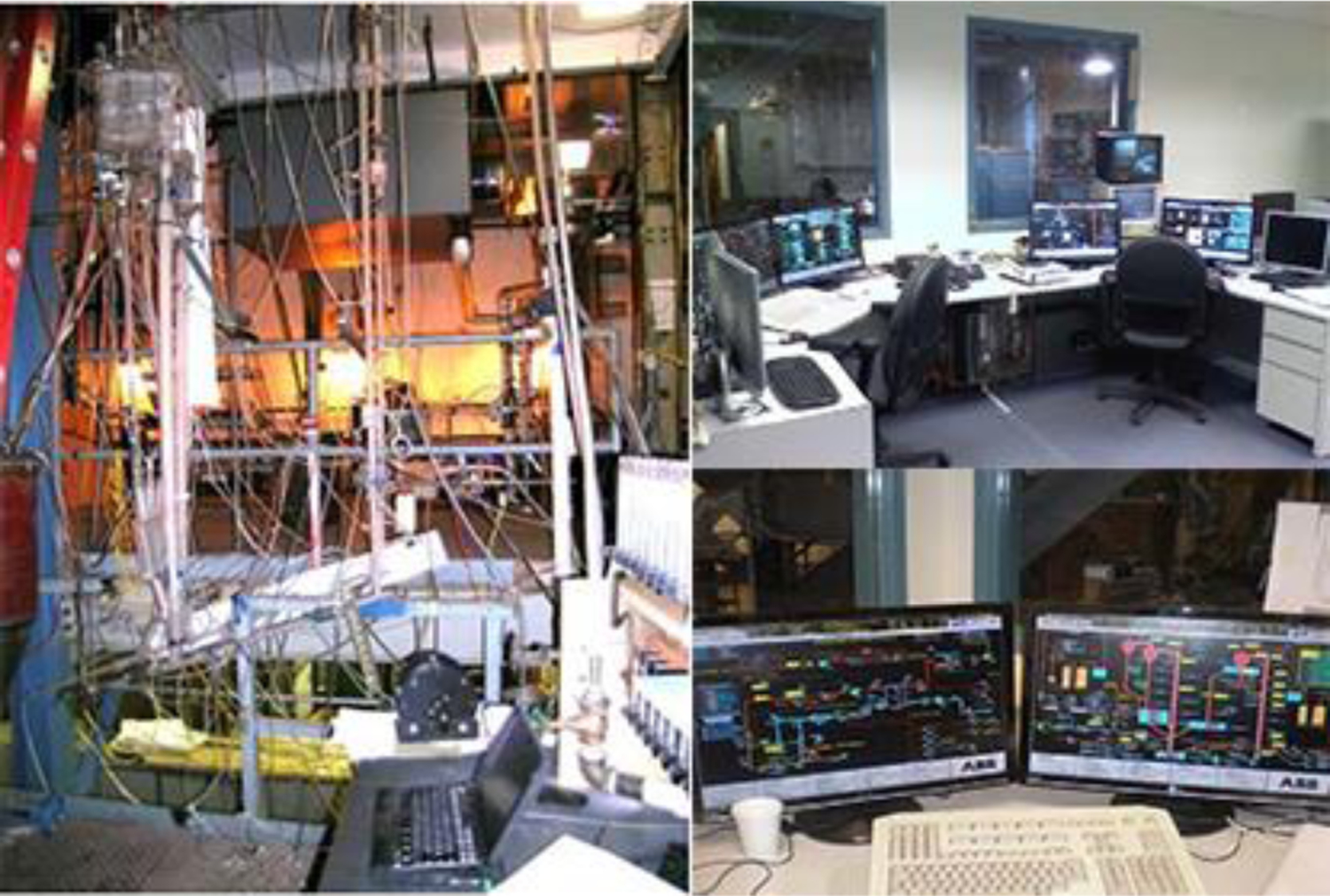
Experimental facility for control testing.

This section describes implementation of the proposed wavelet MRA model-based GPC scheme on the single loop gas/solid cold flow CL process testbed developed at Alstom Power Inc. to carry out experiments without consideration of the oxidation reaction. The experimental facility is shown in Figure 4.

The controllers were developed in MATLAB/SIMULINK, compiled in C and run on the proprietary ASTOM processing platform. The software used for wavelet identification was MATLAB Wavenet. The system output *y* was selected to be the riser pressure drop DP47 (inch H_2_O). Fluidizing air flow *S*1 (standard cubic feet per hour, scfh), was used as the single control input *u*, while the other air flow *S*2 (scfh) was set to a constant value of about 20 scfh. The characterization of the complex dynamic behavior, to be obtained through the identification procedure, was chosen as a SISO NARX wavelet multiresolution network model of the form:

(51) $$\hat{y}\left(t\right)\;=\;f\left(y\left(t\;-\;1\right),\;\cdots \;,\;y\left(t\;-\;n_{y}\right),\;u\left(t-1\right),\;\cdots \;,\;u\left(t\;-\;n_{u}\right)\right)\;=\;\overset{m}{\underset{i=1}{\sum }}\theta_{i}g_{i},$$

where *f* is the unknown nonlinear mapping to be identified, *u*(*t*) and *y*(*t*) are the sampled input and output sequences, *n*_*y*_ and *n*_*u*_ are the maximum lags in the output and the input to be determined, respectively; *θ* = {*θ*_*i*_} is the parameter vector trained on-line, *g*_*i*_ ∈ {*ϕ*_*j*,*k*_, *ψ*_*j*,*k*_} is a multivariable scaling or wavelet basis function of past inputs and outputs, and *m* is the number of required basis functions to meet satisfactory modeling accuracy requirements.

**Figure 5.**
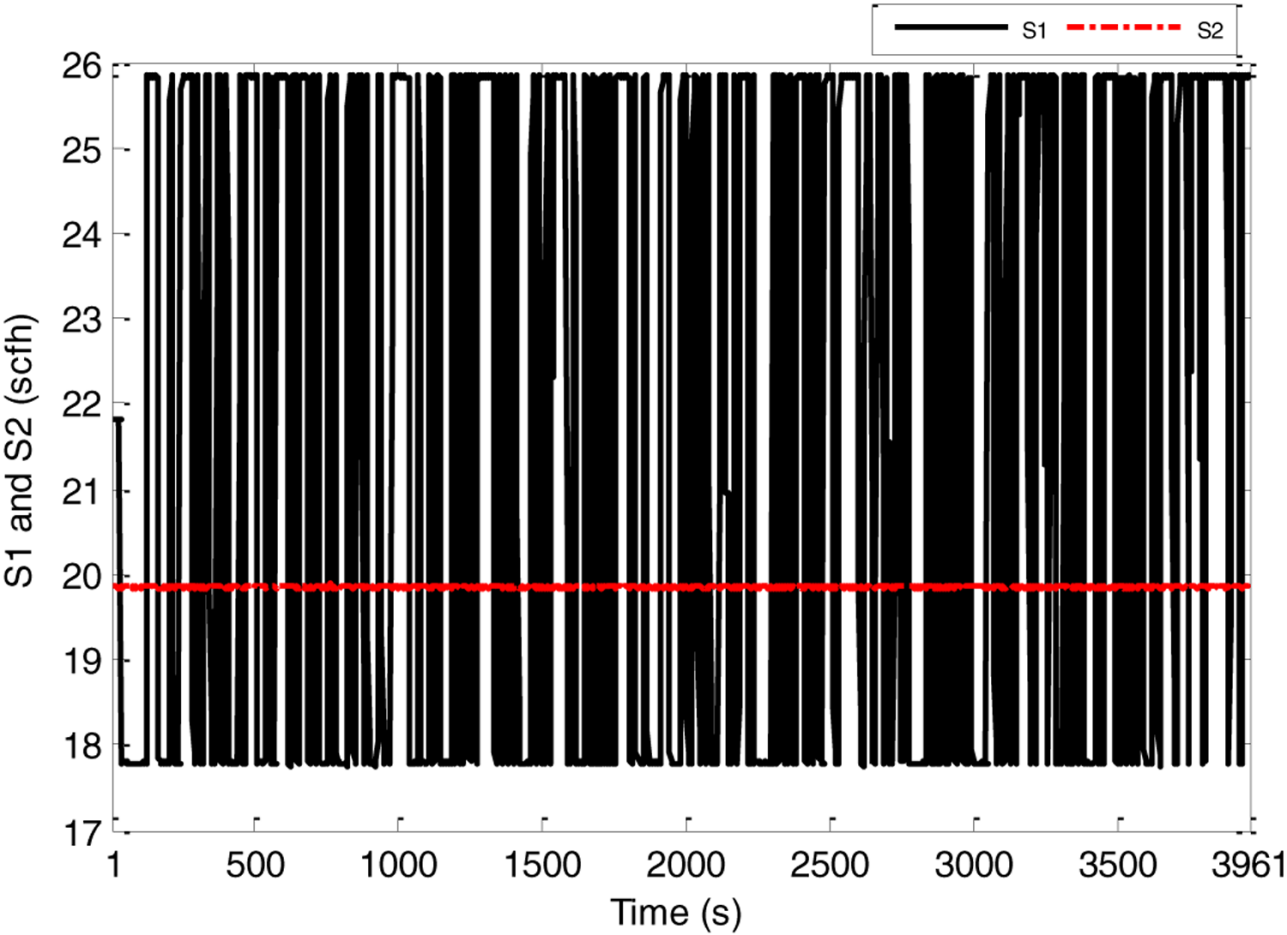
PRBS test-input S1 and S2 (scfh).

**Figure 6.**
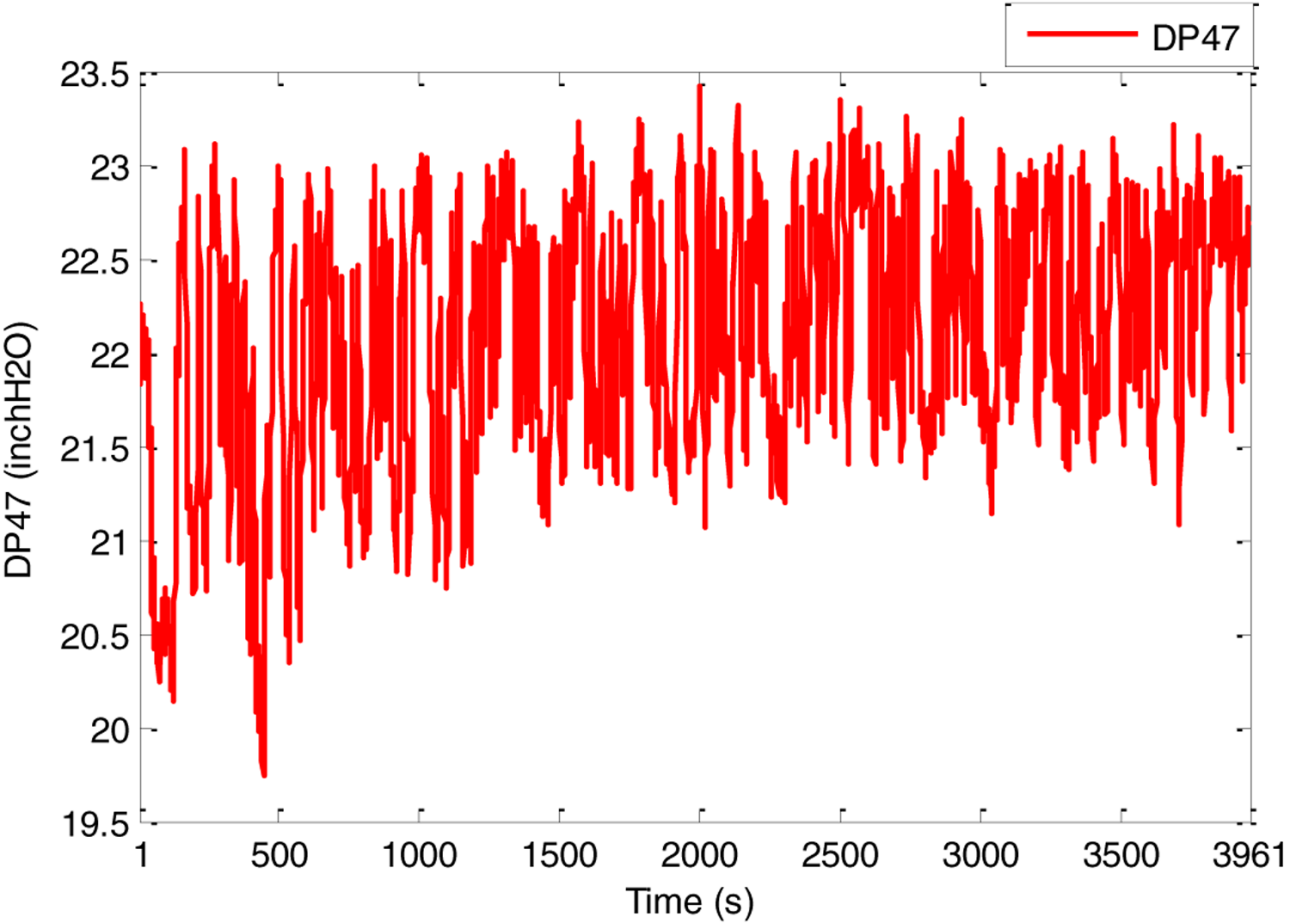
PRBS test-Output DP47 (inch H_2_O).

First, several offline experimental tests were performed to understand the process better and to leverage the test results in tuning the identification structure and the model parameters. The input signal *S*_1_ was generated in the form of a pseudo random binary signal (PRBS) changed about a nominal value, and the pressure drop across the riser DP47 was measured as the output. All the sequences used in the experiment were generated by MATLAB commands. The experimental results of the PRBS test are shown in Figures 5 and 6 below, where the sampling period is 1 s.

The data set consists of 3961 input and output samples. The NARX multiresolution wavelet MRA model was used to approximate the nonlinear relationship between *S*1 and DP47 based on the experimental data. The regressor set was specified as:

(52) y(t−1),y(t−2),u(t−1),u(t−2),⋯,u(t−8).

Hence *n*_*y*_ = 2, *n*_*u*_ = 8. For MRA model we chose radial Marr scaling and wavelet functions [12]:

(53) $$\phi \left(x\right)\;=\;\text{exp}\left(-0.5{\left\|x\right\|}^{2}\right),\;\psi \left(x\right)\;=\;\left(\text{dim}\left(x\right)\;-\;{\left\|x\right\|}^{2}\right)\;\text{exp}\left(-0.5{\left\|x\right\|}^{2}\right).$$

**Figure 7.**
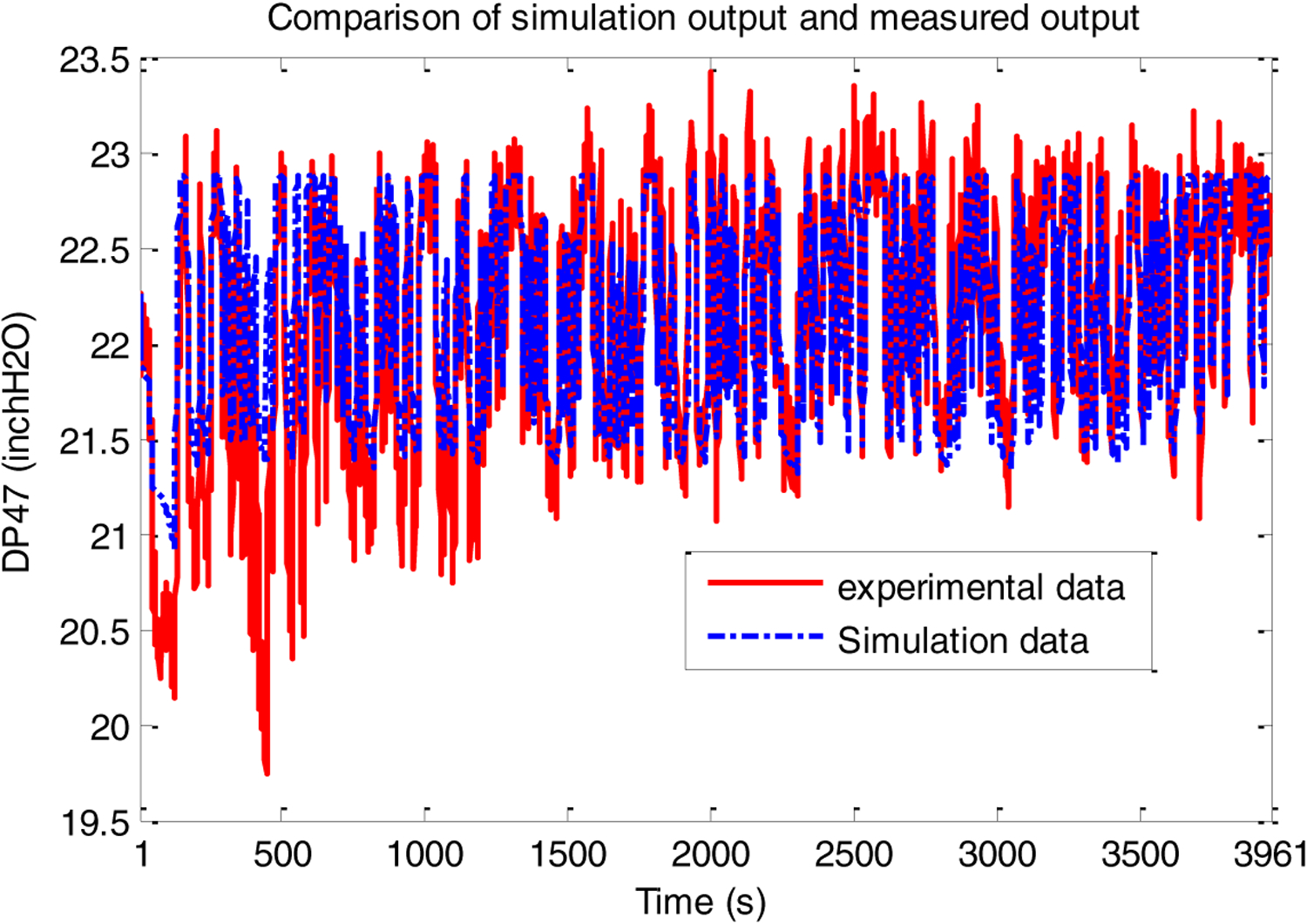
PRBS test-Simulation data vs. experimental data.

The initial coarse layer index *J* was chosen to be 3, with the number of basis functions doubling when resolution was incremented by 1 starting with 10. The final resolution adopted was *K* = 6. Figure 7 below shows how the model predicted output compares with the experimental results.

**Figure 8.**
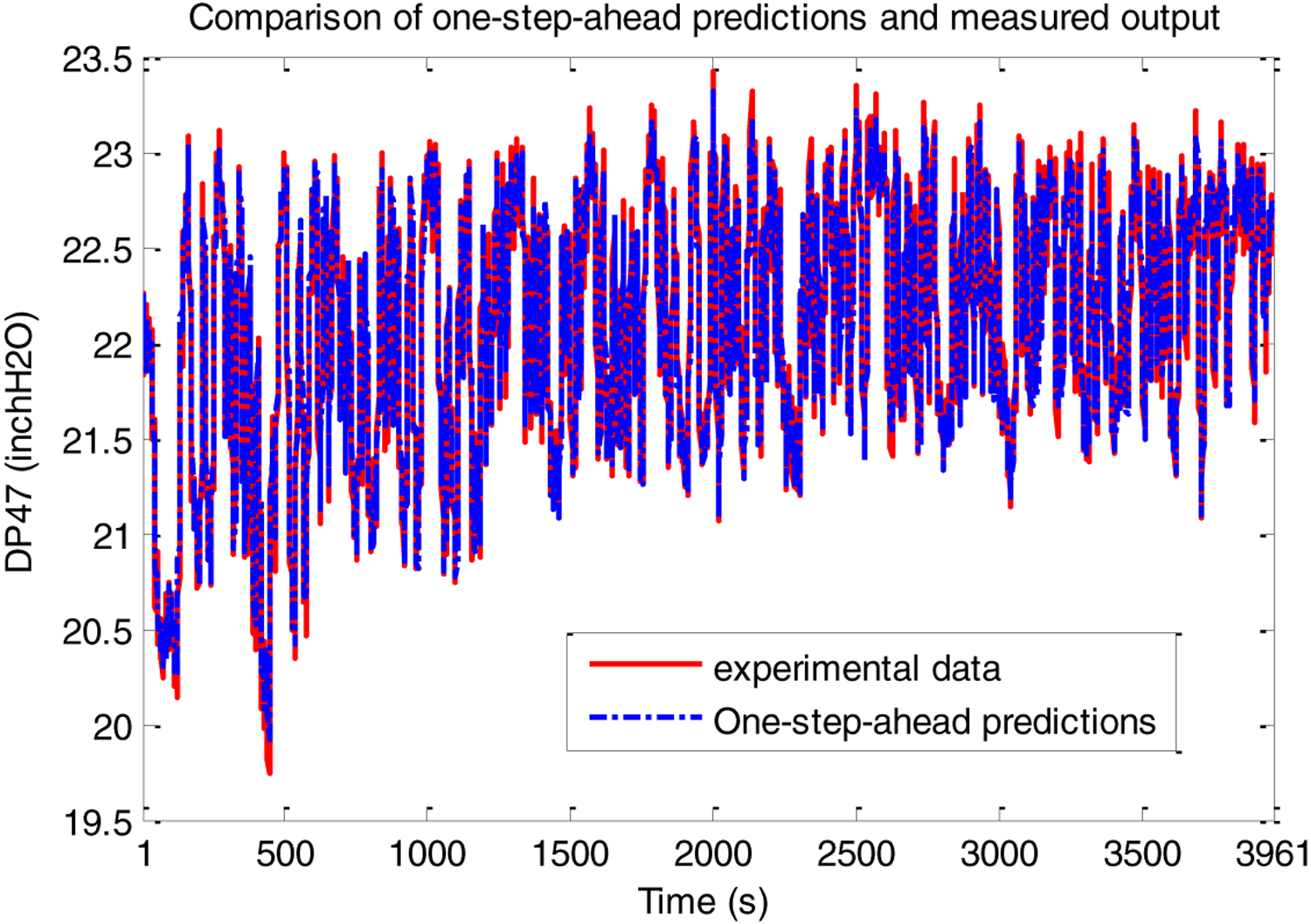
PRBS test-One-step-ahead predictions vs. experimental data.

The one-step-ahead predicted output and the test data set are shown in Figure 8. From these figures it is seen that the NARX wavelet MRA model obtained predicted the system outputs well. The model was found to be sufficiently accurate and no finer resolution levels were needed to be added to the model structure.

### 3.2. MRA–GPC Scheme Implementation on a Chemical Looping Process Testbed

In order to make the wavelet MRA GPC applicable to the CL process, the control input *u* was subjected to rate constraints of the form:

(54) $$\Delta u\;=\;\Delta u_{\text{target}}\;\times \;\exp\left(1-\mu \left\|\Delta u_{\text{target }}\right\|\right),$$

where Δ*u*_target_ is the unconstraint control signal calculated by the predictive control law and *μ* > 0 is a design parameter to adjust the rate of the control signal. The effectiveness of such input constrained wavelet predictive controller on the CL process is demonstrated next through experimental results.

**Figure 9.**
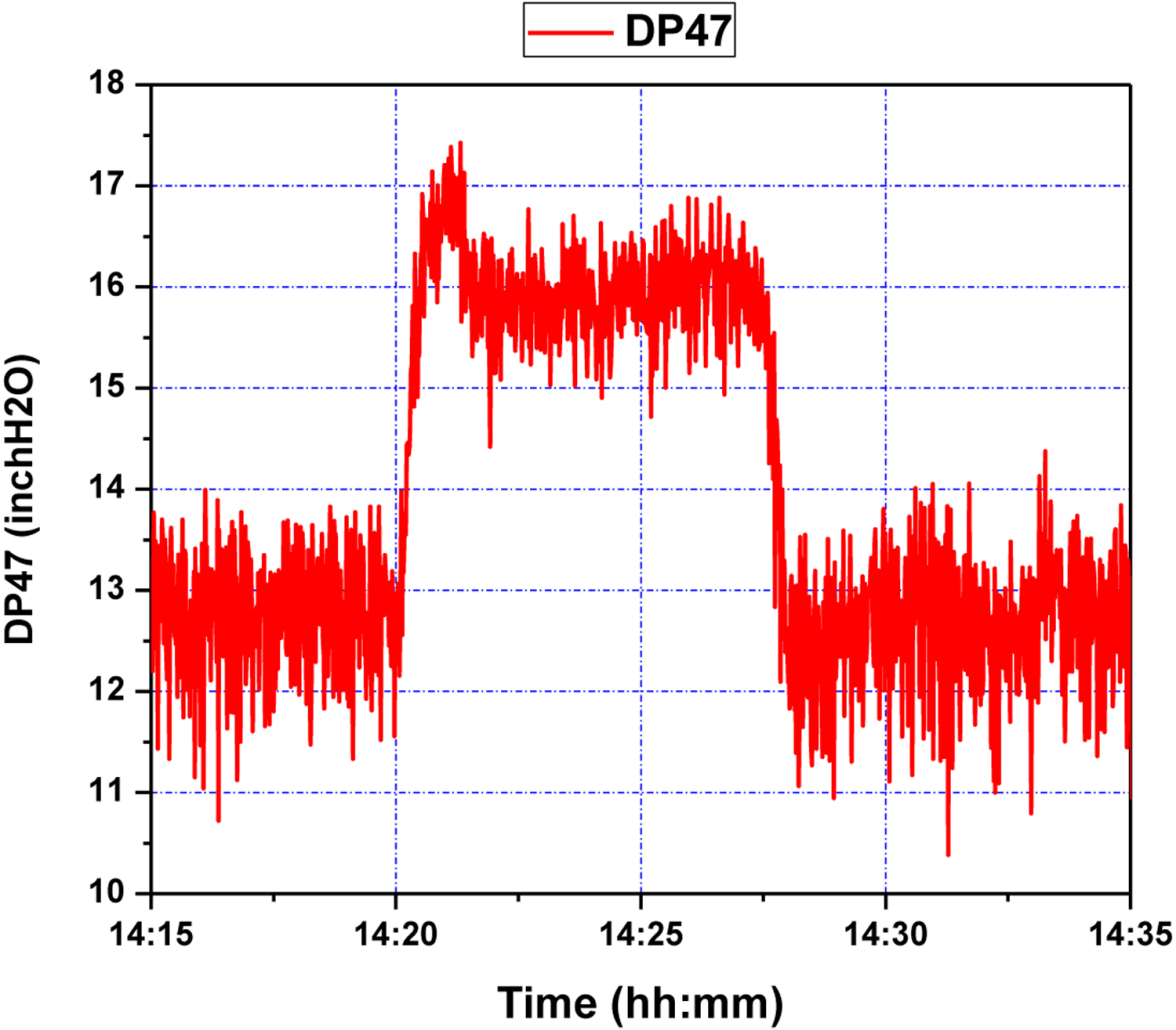
Pressure difference response of riser (DP47) during step setpoint changes.

**Figure 10.**
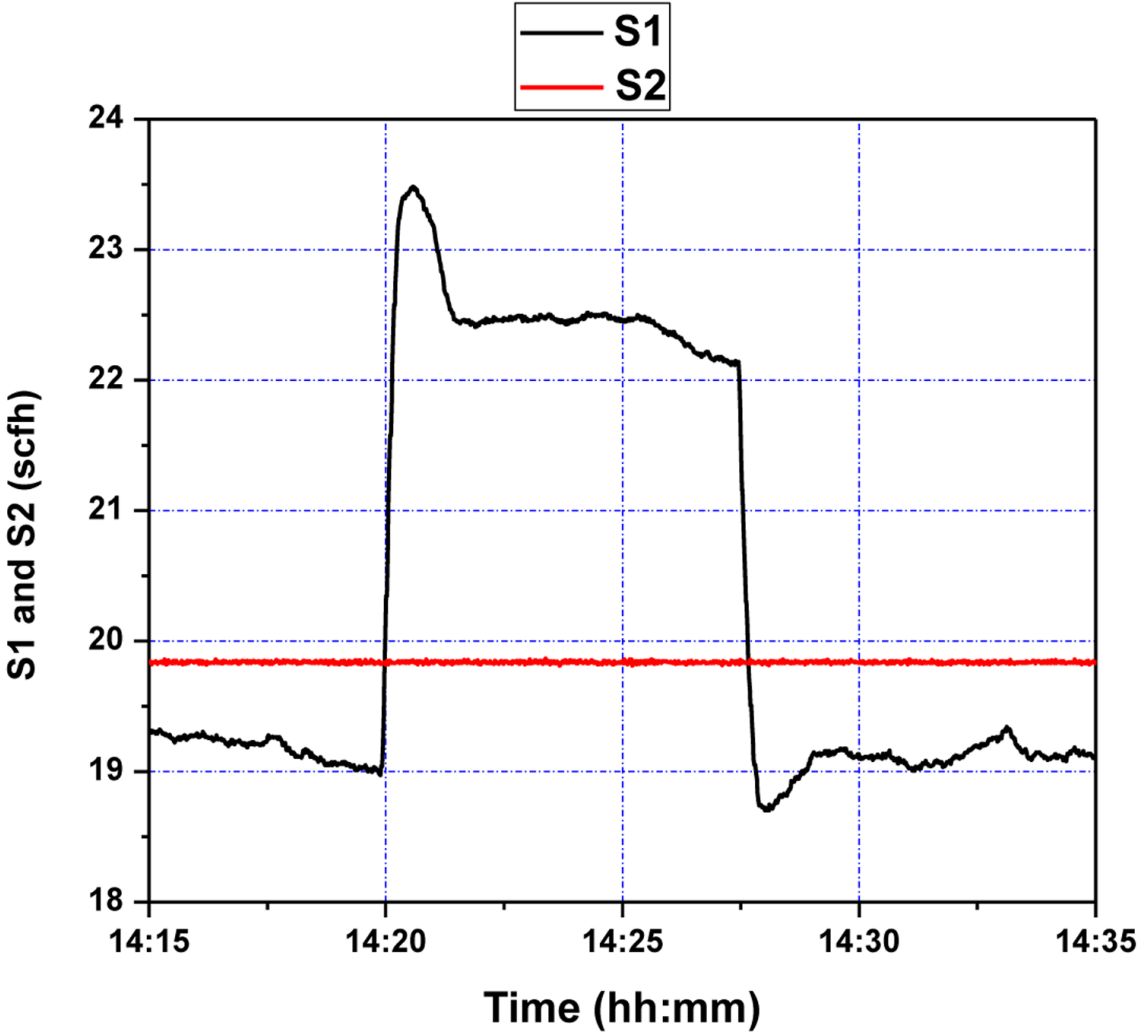
Fluidizing air flow control (S1 and S2) during step setpoint changes.

The control objective of the GPC design is to ensure that the output of the system *y* asymptotically tracks the reference signal *y*_*m*_. The cost function to be minimized is defined in (19). The design parameters for GPC configuration were chosen as *N*_1_ = 1, *N*_2_ = 10, *N*_*u*_ = 8, *ρ* = 1. The adaptation gains derived from Theorems 1 and 2, were chosen as *γ*_*θ*_ = 0.01, *γ*_*u*_ = 0.1. The system was initially stable around level of *y*_0_ = 13 inch H_2_O. Two setpoint step change experiments were performed consecutively. After 5 min, the setpoint was first increased to 16 inch H_2_O and stayed at the latter value for about 7 min. Then it went back to the original level of 13 inch H_2_O. The air flow *S*2 was set to a constant value of about 20 scfh. The tracking response of the system output and the corresponding control efforts are shown in Figures 9 and 10, respectively. It can be seen from these figures that the proposed wavelet MRA-based GPC method effectively tracks the setpoint changes for a single loop CL process.

As can be seen in Figure 9, starting at a setpoint of 13 inH_2_O for the duration of five minutes, responds to a setpoint change at 14:20 within roughly two minutes. While there is an initial overshoot, this overshoot has a magnitude of one inH_2_O, and is quickly subdued; a similar phenomenon is seen at 14:27, when the setpoint is restored to 13 inH_2_O. This points to an adequate response to step-changes in DP47 setpoint, which the process control methodology is capable of handling due to a sufficiently accurate wavelet MRA model, and effective GPC tuning; the latter can be seen in Figure 10, where controller rates are limited to within feasible bounds, and control efforts are limited, making for a subdued control input history.

**Figure 11.**
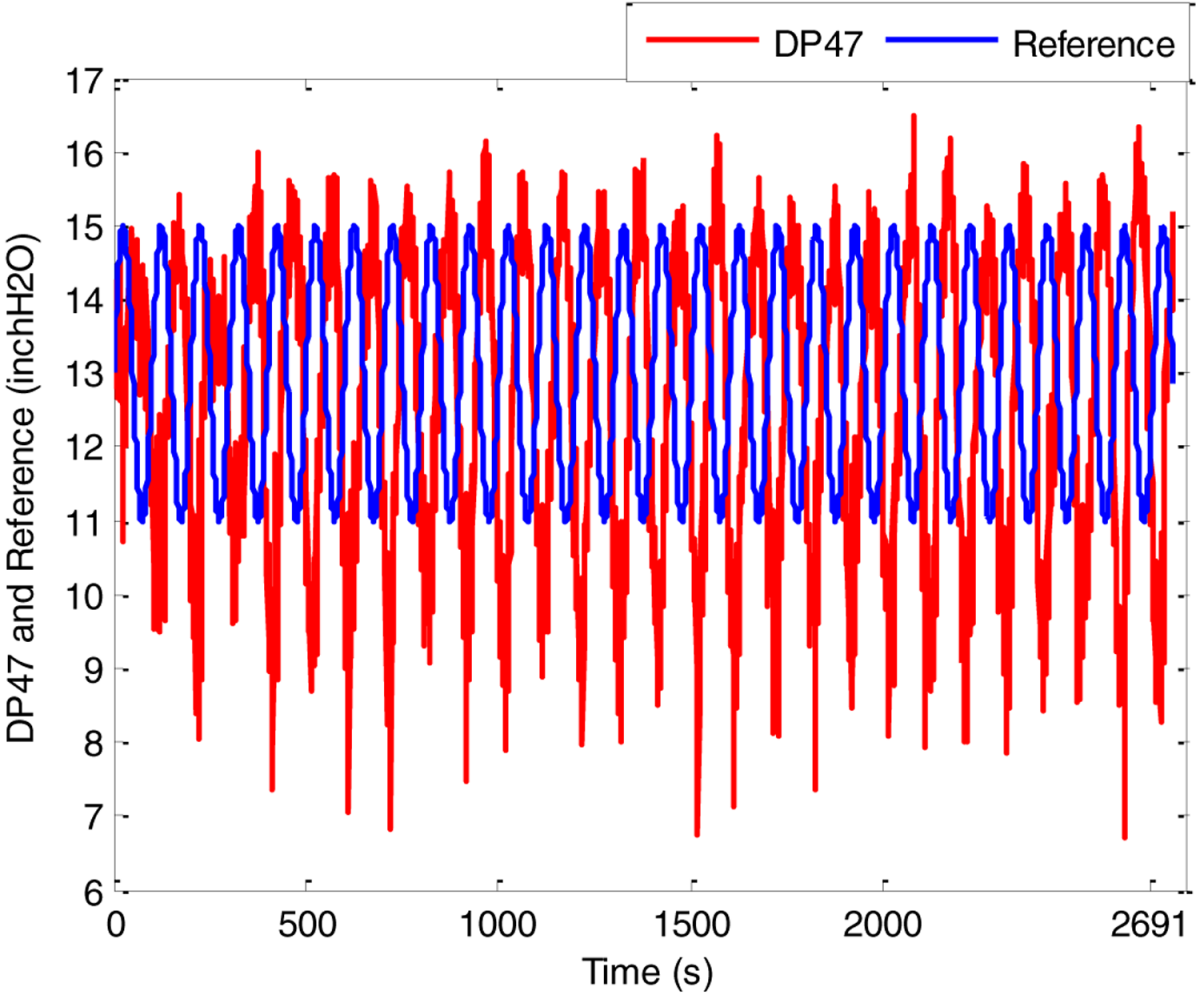
Pressure difference response of riser during sinusoidal setpoint changes.

**Figure 12.**
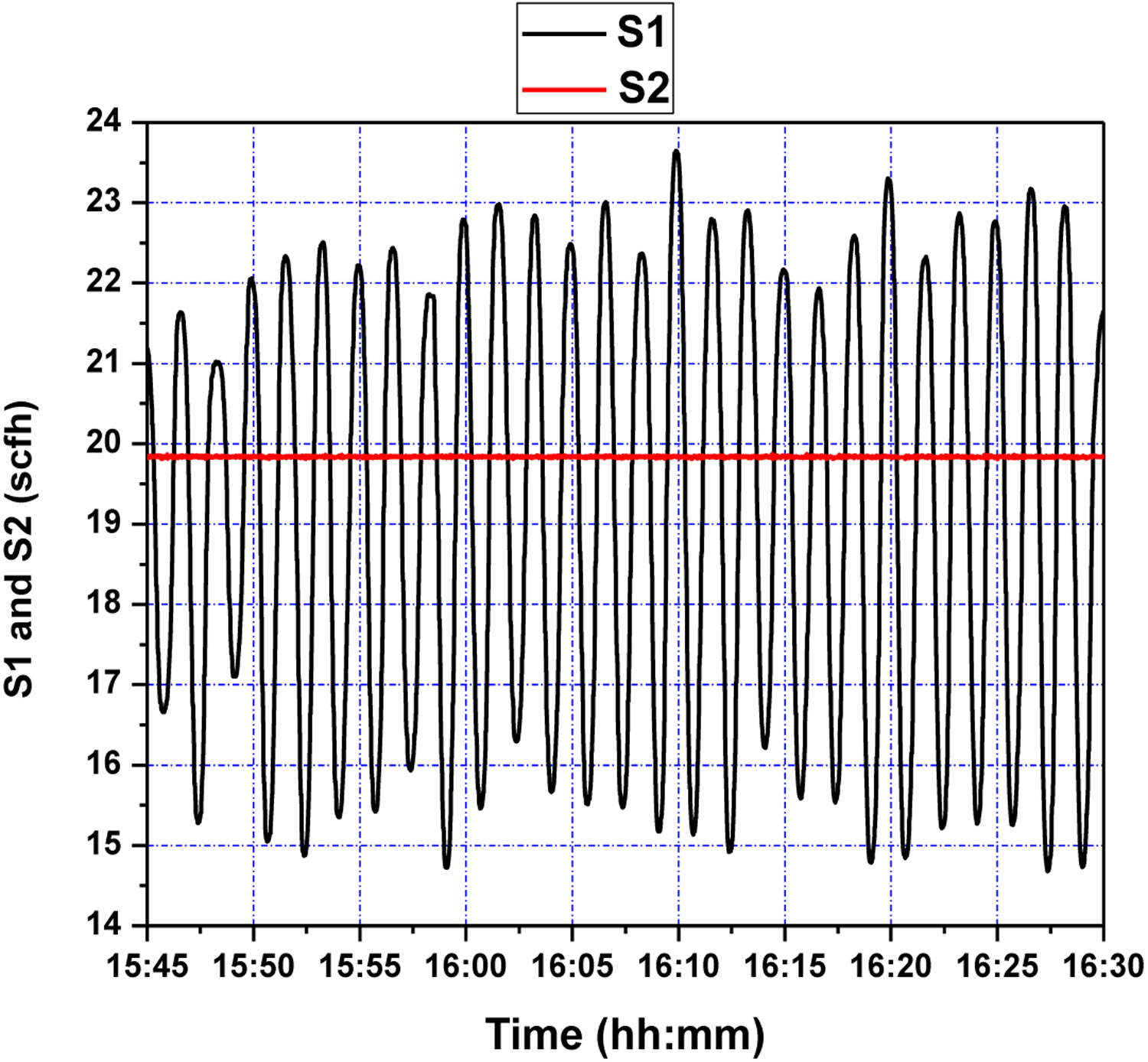
Fluidizing air flow control (S1 and S2) during setpoint changes.

In the second test, reference signal was set to be a sinusoidal of the form *y*_*m*_(*t*) = 13 + 2 sin(2*π* × 0.01 × *t*), while *S*2 (scfh) was still held at a constant value of about 20. The tracking response of system output and the corresponding control effort, shown in Figures 11 and 12, respectively, demonstrate that the controller satisfies the tracking performance requirement, with a time delay between the control signal and system output potentially addressed through prediction adjustment.

In particular, a relatively timely response to reference signal changes is clearly seen in Figure 11; the controller effectuates the reference signal in about 50 s, with little overshoot, as was seen in the previous setpoint step-change experiment. However, besides the phase difference between the reference and true output, large values of undershoot are seen. This can be attributed to the presence of overshoot; as GPC reacts to samples 10 s ahead of time, any overshoot is met with an overaggressive response to lower it (see Figure 12), resulting in excessive undershoot. This issue may be addressed by increasing the control weighing factor to penalize excessive actuation.

The next section presents the derivation and implementation of the spatiotemporal control law for the fast riser dynamics to augment the temporal controller described above and tighten the closed loop tracking performance.

## 4. Spatiotemporal Wavelet Decomposition

**Figure 13.**
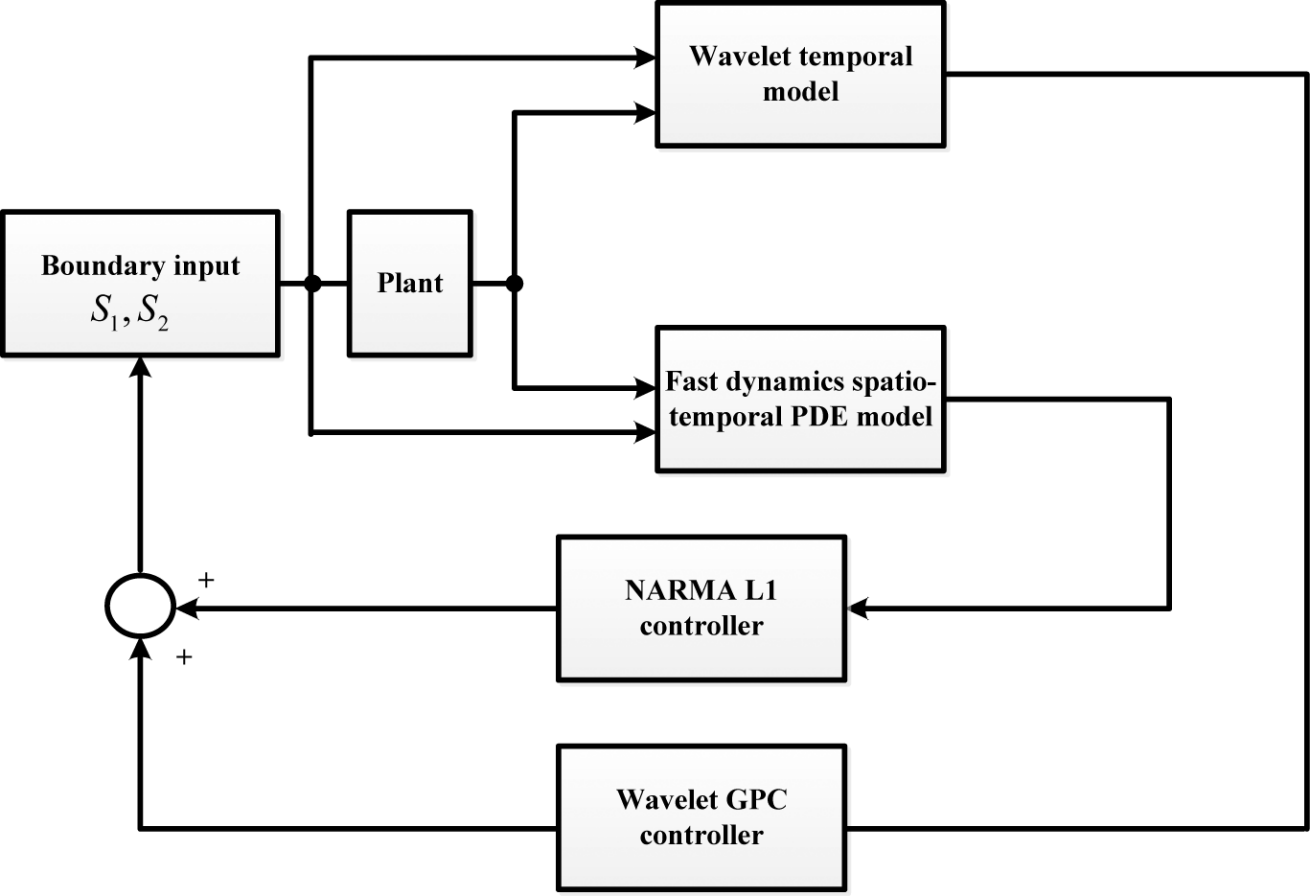
Block diagram of controller implementation with fast dynamics.

Since the empirically identified wavelet temporal model was obtained using data collected at a 1 s sampling rate, some of the fast dynamics of the plant that gave rise to jumps were not recorded. The fast dynamics comes primarily from the spatially distributed riser geometry. Hence, we simulated the impulse response of the 2-PDE riser model [37], approximated the faster riser dynamics with the transient spatiotemporal NARMA-L1 [38] model, and used the result in a convolution to obtain a spatiotemporal model of the transients. We then put the empirical temporal NARX model and the fast dynamics spatiotemporal model in parallel. Finally, we combined the temporal GPC control and the spatiotemporal deadbeat control, each based on its respective model, into the closed loop dual-model self-tuning configuration shown in Figure 13. In this configuration, the dynamic inter-loop coupling is rather minimal due to significantly differing time scales of each elf-tuning loop, ideally requiring two-sampling-rates hardware/software setting, not available for this experiment.

The nonlinear 2-PDE model governing the evolution of the variables (voidage and solid velocity) in the riser can be represented [37] as:

(55) $$\frac{\partial \varepsilon }{\partial t}\;=\;\left(1\;-\;\varepsilon \right)\frac{\partial u_{s}}{\partial x}\;-\;u_{s}\frac{\partial \varepsilon }{\partial x},\;\frac{\partial u_{\text{s}}}{\partial t}\;=\;-u_{s}\frac{\partial u_{\text{s}}}{\partial x}\;+\;C_{1}\varepsilon^{-6.7}\;-\;C_{2}\varepsilon^{-5.7}u_{s}\;+\;C_{3}\varepsilon^{-4.7}u_{\text{S}}^{2}\;+\;C_{4}{\left(1-\varepsilon \right)}^{-0.54}\;-\;C_{5},$$

where *ε* is the voidage and *u*_*s*_ is the solid velocity. The other parameters are defined in [37]. From simulations of this model, we could obtain a response *h*(*x*, *t*) to an impulse actuation in solid velocity with area of 0.1.

**Figure 14.**
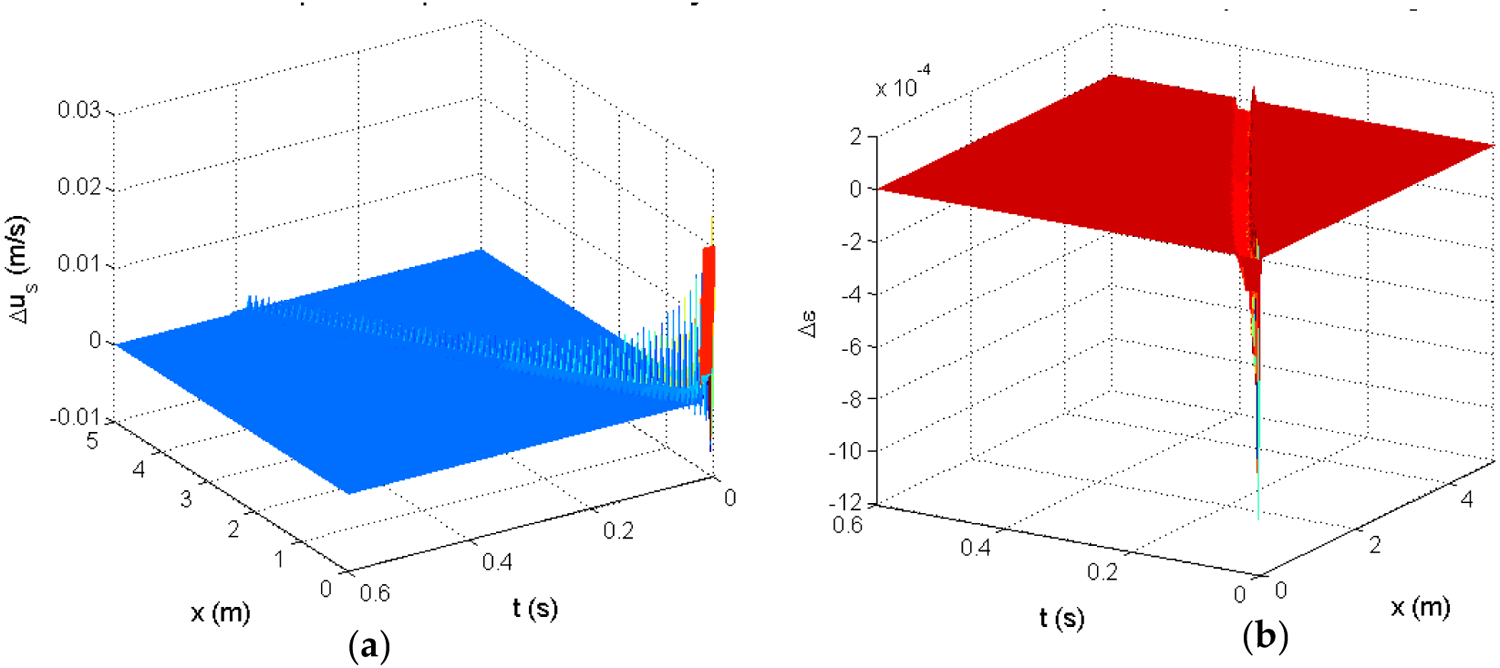
Simulated impulse response of the 2-PDE riser model: (**a**)—the solid velocity response, (**b**)—the voidage response.

Then the response to an arbitrary inlet solid velocity *u*(*t*) can be calculated as:

(56) $$y\left(x,t\right)\;=\;\int_{-\infty }^{t}h\left(x,\tau \right)\cdot 10u\left(x\;-\;\tau \right)d\tau ,$$

where the scaling factor is necessary since the simulated input was not 1. The simulated impulse responses are shown in Figure 14.

Since the impulse response is uniformly zero after 0.6 s, Equation (56) can be limited to:

(57) $$y\left(x,t\right)\;=\;\int_{t-0.6}^{t}h\left(x,\tau \right)\cdot 10u\left(x\;-\;\tau \right)d\tau .$$

To obtain a low-order high fidelity finite-dimensional representation of the impulse response, a wavelet decomposition [39,40] was used to approximate *h*(*x*, *t*). That is, the impulse response *h*(*x*, *t*) was approximated as:

(58) $$h\left(x,t\right)\;=\;\overset{m_{\text{max}}}{\underset{m=1}{\sum }}\overset{n_{\text{max}}}{\underset{n=1}{\sum }}\beta_{m}\left(x\right)c_{m,n}\alpha_{n}\left(t\right),$$

where {*β*_*m*_(*x*)} and {*α*_*n*_(*t*)} are wavelet basis functions.

**Figure 15.**
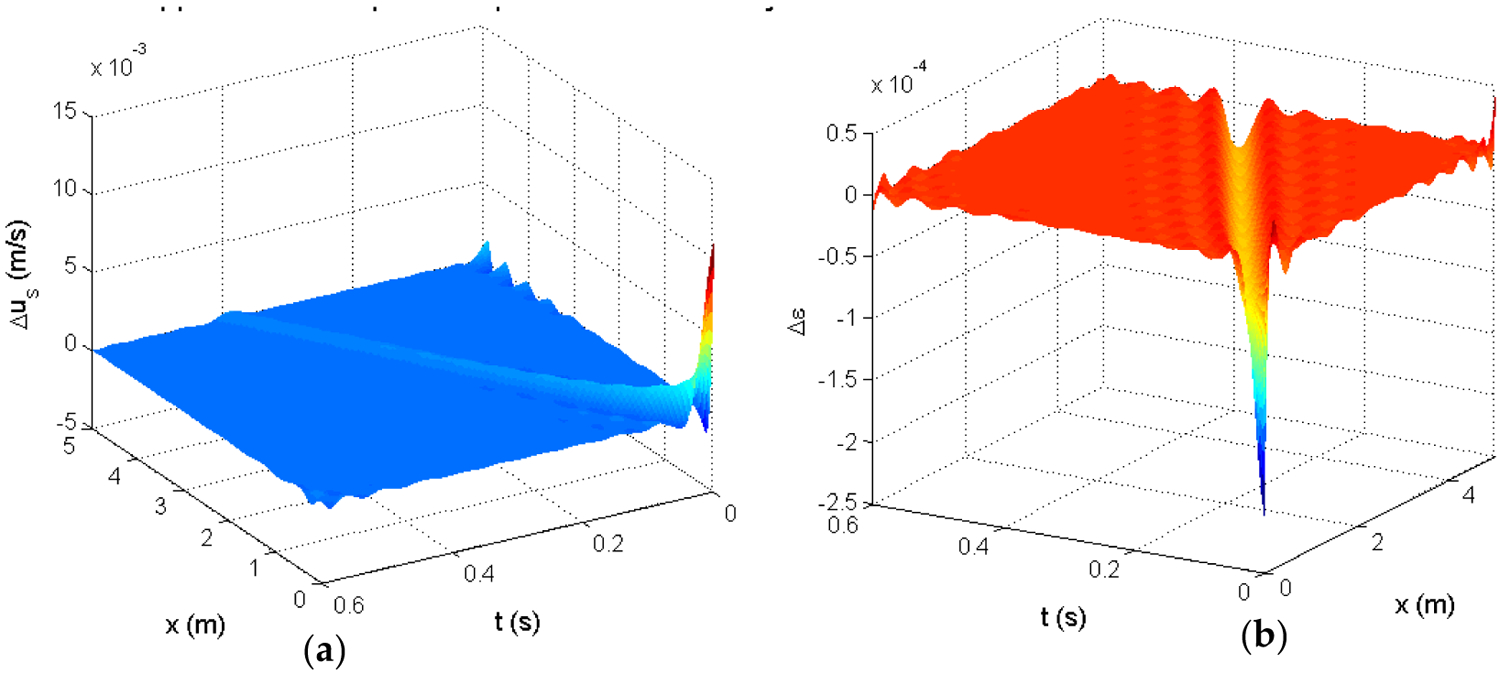
Wavelet-approximated impulse response *h*(*x*, *t*): (**a**)—the solid velocity response approximation, (**b**)—the voidage response approximation.

Figure 15 is the resulting wavelet approximation of the impulse response. Here we chose Gaussian wavelet functions specifically. In this case, 23 spatial and 22 temporal wavelets were used. The coefficients *c*_*m*,*n*_ were determined using a least-squares regression.

The following notation is used to divide the impulse response into separate parts for voidage *ε* and velocity *u*_*s*_:

(59) $$h_{u_{s}}\left(x,t\right)\;=\;\overset{m_{\text{max}}}{\underset{m=1}{\sum }}\overset{n_{\text{max}}}{\underset{n=1}{\sum }}\beta_{m}\left(x\right)c_{m,n}\alpha_{n}\left(t\right),\;h_{\varepsilon }\left(x,t\right)=\overset{m_{\text{max}}}{\underset{m=1}{\sum }}\overset{n_{\text{max}}}{\underset{n=1}{\sum }}\beta_{m}\left(x\right)d_{m,n}\alpha_{n}\left(t\right).$$

Then using the convolution:

(60) $$\Delta u_{s}\left(x,t\right)\;=\;h_{u_{s}}\left(x,\tau \right)\;*\;10\left(u\;-\;\tau \right)\;=\;10\int_{0}^{0.6}\overset{m_{\text{max}}}{\underset{m=1}{\sum }}\overset{n_{\text{max}}}{\underset{n=1}{\sum }}\beta_{m}\left(x\right)c_{m,n}\alpha_{n}\left(\tau \right)u\left(t\;-\;\tau \right)d\tau .$$

Since the online measurements were only available at 1 s intervals, it was assumed that:

(61) u(t−τ)=(1−τ)u(t)+τu(t−1),0≤τ≤1,

to linearly interpolate between the measurements. Then:

(62) $$\Delta u_{s}\left(x,t\right)\;=\;10\int_{0}^{0.6}\sum_{m=1}^{m_{\text{max}}}\sum_{n=1}^{n_{\text{max}}}\beta_{m}c_{m,n}\alpha_{n}\left(\tau \right)\left[\left(1\;-\;\tau \right)u\left(t\right)\;+\;\tau u\left(t\;-\;1\right)\right]d\tau \;=\;10\sum_{m=1}^{m_{\text{max}}}\sum_{n=1}^{n_{\text{max}}}\beta_{m}\left(x\right)c_{m,n}\int_{0}^{0.6}\alpha_{n}\left(\tau \right)\left[\left(1\;-\;\tau \right)u\left(t\right)\;+\;\tau u\left(t\;-\;1\right)\right]d\tau \;=\;10\left[\sum_{m=1}^{m_{\text{max}}}\sum_{n=1}^{n_{\text{max}}}\beta_{m}\left(x\right)c_{m,n}\int_{0}^{0.6}\left(1\;-\;\tau \right)\alpha_{n}\left(\tau \right)d\tau \right]u\left(t\right)\;+\;10\left[\sum_{m=1}^{m_{\max}}\sum_{n=1}^{n_{\max}}\beta_{m}\left(x\right)c_{m,n}\int_{0}^{0.6}\tau \alpha_{n}\left(\tau \right)d\tau \right]u\left(t\;-\;1\right).$$

Denote:

(63) $$\alpha_{n,0}\;=\;\int_{0}^{0.6}\left(1\;-\;\tau \right)\alpha_{n}\left(\tau \right)d\tau ,\;\alpha_{n,1}\;=\;\int_{0}^{0.6}\tau \alpha_{n}\left(\tau \right)d\tau ,$$

and:

(64) $$\gamma_{0}\left(x\right)\;=\;10\overset{m_{\text{max}}}{\underset{m=1}{\sum }}\overset{n_{\text{max}}}{\underset{n=1}{\sum }}\beta_{m}\left(x\right)c_{m,n}\alpha_{n,0},\;\gamma_{1}\left(x\right)\;=\;10\overset{m_{\text{max}}}{\underset{m=1}{\sum }}\overset{n_{\text{max}}}{\underset{n=1}{\sum }}\beta_{m}\left(x\right)c_{m,n}\alpha_{n,1}.$$

Then Equation (62) simplifies to:

(65) $$\Delta u_{s}\left(x,t\right)\;=\;\gamma_{0}\left(x\right)u\left(t\right)\;+\;\gamma_{1}\left(x\right)u\left(t\;-\;1\right).$$

Similarly, we have:

(66) $$\Delta \varepsilon \left(x,t\right)\;=\;\eta_{0}\left(x\right)u\left(t\right)\;+\;\eta_{1}\left(x\right)u\left(t\;-\;1\right),$$

where:

(67) $$\eta_{0}\left(x\right)\;=\;10\overset{m_{\text{max}}}{\underset{m=1}{\sum }}\overset{n_{\text{max}}}{\underset{n=1}{\sum }}\beta_{m}\left(x\right)d_{m,n}\alpha_{n,0},\;\eta_{1}\left(x\right)\;=\;10\overset{m_{\text{max}}}{\underset{m=1}{\sum }}\overset{n_{\text{max}}}{\underset{n=1}{\sum }}\beta_{m}\left(x\right)d_{m,n}\alpha_{n,1}.$$

Omitting for brevity several routine manipulations (available in [23]), the output DP47 representing the pressure drop across the riser can be given as:

(68) $$P\left(5\right)\;-\;P\left(0\right)\;=\;\left[\rho_{g}\varepsilon \left(0\right)u_{g}^{2}\left(0\right)\;+\;\rho_{s}\left(1-\varepsilon \left(0\right)\right)u_{s}^{2}\left(0\right)\right]\;-\left[\rho_{g}\varepsilon \left(5\right)u_{g}^{2}\left(5\right)\;+\;\rho_{s}\left(1-\varepsilon \left(5\right)\right)u_{s}^{2}\left(5\right)\right]\;-\int_{0}^{5}g\left[\rho_{g}\varepsilon \left(y\right)\;+\;\rho_{s}\left(1-\varepsilon \left(y\right)\right)\right]dy,$$

where the riser length of 5 m is used, constant *g* is the gravity acceleration, *u* is the velocity, *ρ* is the density, the subscripts *s* and *g* refer to solid and gas, respectively, and $u_{g}\;=\;\frac{U_{g}}{\varepsilon \left(x\right)}$ where *U*_*g*_ is the superficial gas velocity. Expanding Equation (68) yields:

(69) $$P\left(5\right)\;-\;P\left(0\right)\;=\;\left[\rho_{g}\frac{U_{g}^{2}}{\varepsilon \left(0\right)}\;+\;\rho_{s}\left(1-\varepsilon \left(0\right)\right)u_{s}^{2}\left(0\right)\right]\;-\left[\rho_{g}\frac{U_{g}^{2}}{\varepsilon \left(5\right)}\;+\;\rho_{s}\left(1\;-\;\varepsilon \left(5\right)\right)u_{s}^{2}\left(5\right)\right]\;-\int_{0}^{5}g\left[\rho_{g}\varepsilon (y)\;+\;\rho_{s}\left(1\;-\;\varepsilon \left(y\right)\right)\right]dy\;=\rho_{g}\frac{U_{g}^{2}}{\varepsilon_{ss}\left(0\right)\;+\;\Delta \varepsilon \left(0\right)}\;+\;\rho_{s}\left(1\;-\;\varepsilon_{ss}\left(0\right)\;-\;\Delta \varepsilon \left(0\right)\right){\left(u_{s}\left(0\right)\;+\;\Delta u_{s}\left(0\right)\right)}^{2}\;-\rho_{g}\frac{U_{g}^{2}}{\varepsilon_{ss}\left(5\right)\;+\;\Delta \varepsilon \left(5\right)}\;-\;\rho_{s}\left(1\;-\;\varepsilon_{ss}\left(5\right)\;-\;\Delta \varepsilon \left(5\right)\right){\left(u_{s}\left(5\right)\;+\;\Delta u_{s}\left(5\right)\right)}^{2}\;-g\rho_{g}\int_{0}^{5}\left(\varepsilon_{ss}\left(y\right)\;+\;\Delta \varepsilon \left(y\right)\right)dy\;-\;g\rho_{s}\int_{0}^{5}\left(1\;-\;\varepsilon_{ss}\left(y\right)\;-\;\Delta \varepsilon \left(y\right)\right)dy,$$

where the subscript *ss* designates the steady state. Now, substituting the wavelet model gives:

(70) $$P\left(5\right)\;-\;P\left(0\right)\;=\;\rho_{g}\frac{U_{g}^{2}}{\varepsilon_{ss}\left(0\right)\;+\;\eta_{0}\left(0\right)u\left(t\right)\;+\;\eta_{1}\left(0\right)u\left(t\;-\;1\right)}\;+\rho_{s}\left[1\;-\;\varepsilon_{ss}\left(0\right)\;-\;\eta_{0}\left(0\right)u\left(t\right)\;-\;\eta_{1}\left(0\right)u\left(t\;-\;1\right)\right]{\left[u_{s}\left(0\right)\;+\;\gamma_{0}\left(0\right)u\left(t\right)\;+\;\gamma_{1}\left(0\right)u\left(t\;-\;1\right)\right]}^{2}\;-\rho_{g}\frac{U_{g}^{2}}{\varepsilon_{ss}\left(5\right)+\eta_{0}\left(5\right)u\left(t\right)+\eta_{1}\left(5\right)u\left(t\;-\;1\right)}\;-\rho_{s}\left[1-\varepsilon_{ss}\left(5\right)\;-\;\eta_{0}\left(5\right)u(t)\;-\;\eta_{1}\left(5\right)u\left(t\;-\;1\right)\right]{\left[u_{s}\left(5\right)\;+\;\gamma_{0}\left(5\right)u\left(t\right)\;+\;\gamma_{1}\left(5\right)u\left(t\;-\;1\right)\right]}^{2}\;-g\rho_{g}\int_{0}^{5}\left(\varepsilon_{ss}\left(y\right)\;+\;\eta_{0}\left(y\right)u\left(t\right)\;+\;\eta_{1}\left(y\right)u\left(t\;-\;1\right)\right)dy\;-g\rho_{s}\int_{0}^{5}\left(1\;-\;\varepsilon_{ss}\left(y\right)\;-\;\eta_{0}\left(y\right)u\left(t\right)\;-\;\eta_{1}\left(y\right)u\left(t\;-\;1\right)\right)dy.$$

Our goal was to use the model given by Equation (70) to account for the spatiotemporal behavior of the CL system to the extent allowed by the available sampling rate, and also to develop the control setting to be ready to employ much higher sampling rates for performance improvement, once they become available on the test rig through the processor upgrades to the GPUs and FPGAs. It is also of interest to calculate the steady-state pressure drop:

(71) $$\Delta P_{0}\;=\;\rho_{g}\frac{U_{g}^{2}}{\varepsilon_{ss}\left(0\right)\;+\;\eta_{0}\left(0\right)u\left(t\right)\;+\;\eta_{1}\left(0\right)u\left(t\right)}\;+\rho_{s}\left[1\;-\;\varepsilon_{ss}\left(0\right)\;-\;\eta_{0}\left(0\right)u\left(t\right)\;-\;\eta_{1}\left(0\right)u\left(t\right)\right]{\left[u_{s}\left(0\right)\;+\;\gamma_{0}\left(0\right)u\left(t\right)\;+\;\gamma_{1}\left(0\right)u\left(t\right)\right]}^{2}\;-\rho_{g}\frac{U_{g}^{2}}{\varepsilon_{ss}\left(5\right)\;+\;\eta_{0}\left(5\right)u\left(t\right)\;+\;\eta_{1}\left(5\right)u\left(t\right)}\;-\rho_{s}\left[1\;-\;\varepsilon_{ss}(5)\;-\;\eta_{0}\left(5\right)u\left(t\right)\;-\;\eta_{1}\left(5\right)u\left(t\right)\right]{\left[u_{s}\left(5\right)\;+\;\gamma_{0}\left(5\right)u\left(t\right)\;+\;\gamma_{1}\left(5\right)u\left(t\right)\right]}^{2}\;-g\rho_{g}\int_{0}^{5}\left(\varepsilon_{ss}\left(y\right)\;+\;\eta_{0}\left(y\right)u\left(t\right)\;+\;\eta_{1}\left(y\right)u\left(t\right)\right)dy\;-g\rho_{s}\int_{0}^{5}\left(1\;-\;\varepsilon_{ss}\left(y\right)\;-\;\eta_{0}\left(y\right)u\left(t\right)\;-\;\eta_{1}\left(y\right)u\left(t\right)\right)dy.$$

This is the pressure drop predicted by this model for constant input *u*(*t*), as opposed to the linear interpolation described above. We can then use this model to approximate the transient difference, and the NARX wavelet model to approximate the steady state. The difference between the transient pressure drop Δ*P*(*t*) and the eventual steady pressure drop Δ*P*_0_(*t*) is then equal to:

(72) $$\Delta P\;-\;\Delta P_{0}\;=\;P\left(5\right)\;-\;P\left(0\right)\;-\;\Delta P_{0}.$$

Linearizing Equation (72) about *u*(*t*) = *u*(*t* − 1) gives:

(73) $$\Delta P\;-\;\Delta P_{0}\;\approx \;f\left(u\left(t\;-\;1\right)\right)\left(u\left(t\right)-u\left(t\;-\;1\right)\right),$$

where:

(74) $$f\left(y\right)\;=\;k_{1}\frac{\left(k_{2}\;+\;k_{3}y\right)}{{\left(k_{2}\;+\;k_{4}y\right)}^{4}}\;+\;k_{5}{\left(k_{6}\;+\;k_{7}y\right)}^{2}\;+k_{8}\left(1\;-\;k_{2}\;-\;k_{4}y\right)\left(k_{6}\;+\;k_{9}y\right)\;+\;k_{10}\frac{\left(k_{11}\;+\;k_{12}y\right)}{{\left(k_{11}\;+\;k_{13}y\right)}^{4}}\;+k_{14}{\left(k_{15}\;+\;k_{16}y\right)}^{2}\;+\;k_{17}\left(1\;-\;k_{11}\;-\;k_{13}y\right)\left(k_{15}\;+\;k_{18}y\right)\;+\;k_{19},$$

and:

(75) $$k_{1}\;=\;\rho_{g}U_{g}\eta_{1}\left(0\right),\;k_{2}\;=\;\varepsilon_{ss}\left(0\right),\;k_{3}=\eta_{0}\left(0\right)\;+\;2\eta_{1}\left(0\right),\;k_{4}\;=\;\eta_{0}\left(0\right)\;+\;\eta_{1}\left(0\right),\;k_{5}\;=\;\rho_{s}\eta_{1}\left(0\right),\;k_{6}\;=\;u_{s,ss}\left(0\right),\;k_{7}\;=\;\gamma_{0}\left(0\right)\;+\;\gamma_{1}\left(0\right),\;k_{8}\;=\;-\rho_{s}\gamma_{1}\left(0\right),\;k_{9}\;=\;\gamma_{0}\left(0\right)\;+\;2\gamma_{1}\left(0\right),\;k_{10}\;=\;-\rho_{g}U_{g}\eta_{1}\left(5\right),\;k_{11}\;=\;\varepsilon_{ss}\left(5\right),\;k_{12}\;=\;\eta_{0}\left(5\right)\;+\;2\eta_{1}\left(5\right),\;k_{13}\;=\;\eta_{0}\left(5\right)\;+\;\eta_{1}\left(5\right),\;k_{14}\;=\;-\rho_{s}\eta_{1}\left(5\right),\;k_{15}\;=\;u_{s,ss}\left(5\right),\;k_{16}\;=\;\gamma_{0}\left(5\right)\;+\;\gamma_{1}\left(5\right),\;k_{17}\;=\;\rho_{s}\gamma_{1}\left(5\right),\;k_{18}\;=\;\gamma_{0}\left(5\right)\;+\;2\gamma_{1}\left(5\right),\;k_{19}\;=\;g\left(\rho_{g}\;-\;\rho_{s}\right)\int_{0}^{5}\eta_{1}\left(y\right)dy.$$

The input to the computational model was in terms of the velocity boundary condition, so that *u*(*t*) = Δ*u*_*s*_(0, *t*). This can be connected to the inputs *S*_1_ and *S*_2_ via a quadratic model fitted to the test data where:

(76) $$u\left(t\right)\;\approx \;\frac{1}{\varepsilon_{0}}\left(2a_{1}S_{1}\left(t\;-\;1\right)\;+\;a_{3}S_{2}\left(t\right)\;+\;a_{4}\right)\left(S_{1}\left(t\right)\;-\;S_{1}\left(t\;-\;1\right)\right)\;+\frac{1}{\varepsilon_{0}}\left(a_{1}S_{1}^{2}\left(t\;-\;1\right)\;+\;a_{2}S_{2}^{2}\left(t\right)\;+\;a_{3}S_{1}\left(t\;-\;1\right)S_{2}\left(t\right)\;+\;a_{4}S_{1}\left(t\;-\;1\right)\;+\;a_{5}S_{2}\left(t\right)\;+\;a_{6}\right)\;-\;u_{s,ss}(0)\;=\frac{1}{\varepsilon_{0}}\left(a_{1}S_{1}\left(t\;-\;1\right)\;+\;a_{3}S_{2}\left(t\right)\;+\;a_{4}\right)S_{1}\left(t\right)\;+\frac{1}{\varepsilon_{0}}\left(a_{2}S_{2}^{2}\left(t\right)\;+\;a_{5}S_{2}\left(t\right)\;+\;a_{6}\right)\;-\;u_{s,ss}\left(0\right).$$

Then:

(77) $$\Delta P\;-\;\Delta P_{0}\;\approx \;\frac{1}{\varepsilon_{0}}f\left(u\left(t\;-\;1\right)\right)\left(2a_{1}S_{1}\left(t\;-\;1\right)\;+\;a_{3}S_{2}\left(t\right)\;+\;a_{4}\right)S_{1}\left(t\right)\;+f\left(u\left(t\;-\;1\right)\right)\left[\frac{1}{\varepsilon_{0}}\left(a_{2}S_{2}^{2}\left(t\right)\;+\;a_{5}S_{2}\left(t\right)\;+\;a_{6}\right)\;-\;u_{s,ss}\left(0\right)\;-\;u\left(t\;-\;1\right)\right]\;=g_{\Delta P}\left(S_{1}\left(t\;-\;1\right),\;S_{2}\left(t\;-\;1\right),\;S_{2}\left(t\right)\right)S_{1}\left(t\right)\;+\;f_{\Delta P}\left(S_{1}\left(t\;-\;1\right),\;S_{2}\left(t\;-\;1\right),S_{2}\left(t\right)\right).$$

The NARX wavelet MRA model takes the form as in Equation (51):

(78) $$y_{w}\left(t\right)\;=\;f\left(y\left(t\;-\;1\right),\;\cdots \;,\;y\left(t\;-\;n_{y}\right),\;S_{1_{-}w}\left(t\;-\;1\right),\;\cdots \;,\;S_{1_{-}w}\left(t-n_{u}\right)\right),$$

where *S*_1,*w*_(*t*) is the control command calculated by wavelet adaptive GPC control. Then, the fast transient behavior model of Equation (77) can be combined with Equation (78) to obtain a spatiotemporal multiscale dynamic network representation of the entire CL process:

(79) $$y\left(t\right)\;\approx \;y_{w}\left(t\right)\;-\;f_{\Delta P}\;-\;g_{\Delta P}S_{1}\left(t\right).$$

The sign change is necessary because the pressure drop across the riser is negative in the model above, i.e., *P*(5) − *P*(0) < 0. Then, the deadbeat predictive controller taking account of fast dynamics is:

(80) $$S_{1,\text{fast}}\left(t\right)\;=\;\frac{y_{r}\left(t\right)\;-\;y_{w}\left(t\right)\;+\;f_{\Delta P}}{g_{\Delta P}},$$

where *y*_*r*_(*t*) is the reference signal. Hence, the final spatiotemporal wavelet controller *S*_1_(*t*) implemented on the real CL process is given by:

(81) $$S_{1}\left(t\right)\;=\;S_{1,w}\left(t\right)\;+\;S_{1,\text{ fast}}\left(t\right).$$

**Figure 16.**
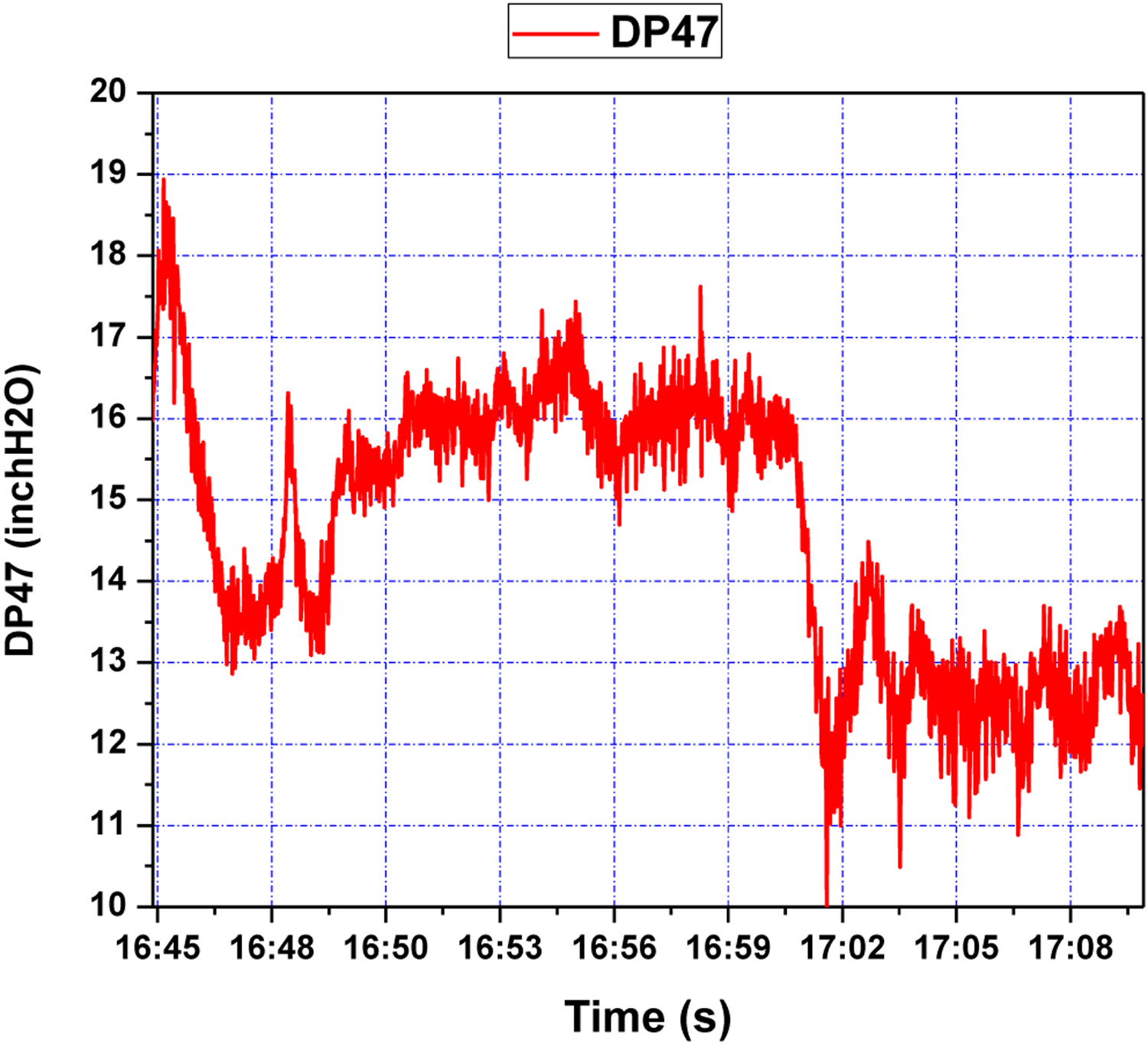
Pressure difference response of riser (DP47) during step setpoint changes.

**Figure 17.**
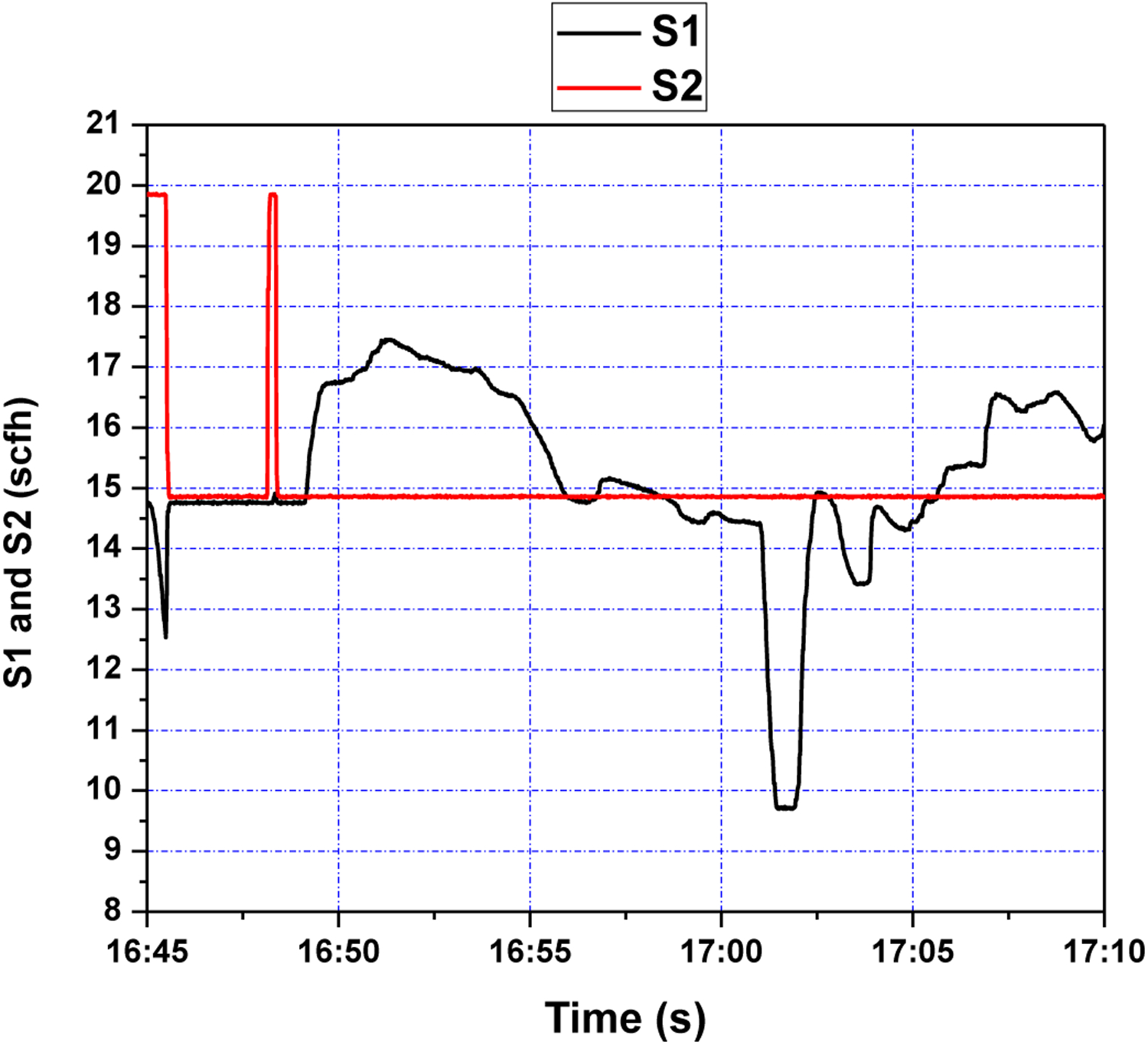
Fluidizing air flow control (S1 and S2) during setpoint changes.

This controller was also implemented in the single loop cold flow CL test rig. *S*_1_ was taken as the single input and DP47 as the output, while *S*_2_ was mostly steady, but with jumps. The reference signal was set to 16 initially and then reduced to 13 around time 17:01. The tracking response of system output and the corresponding control efforts are shown in Figures 16 and 17, respectively. The controller is seen to stabilize the system quite well under difficult operating conditions. The pressure drop DP47 over the riser related to the fluidizing air flows was discussed based on the closed loop topology augmentation with the spatiotemporal model-based control.

## 5. Discussion

The control objective of developing the tracking controller for the CL test rig at ALSTOM Power was addressed at the start of the project through both first-principles model development and empirical system identification from the input/output experimental data record. The first approach resulted in the analytical development and the numerical simulation of the 1D, 2D, and 3D partial differential equation (PDE) networks—systems of coupled PDEs, each describing the testbed subsystem. On the basis of these models, approximately tuned to the process through the experimental data, first, the linear finite dimensional models were developed, after which robust controllers based on the *H*_∞_ approach were designed. The latter were implemented on the test rig; however, no satisfactory performance was obtained. The empirical approach initially involved a polynomial NARX model test fit with the use of model predictive control (MPC) with constraints attained through the QP (quadratic programming) based control signal calculation. At the same time the hypothesis was posed that the process is highly multiscale and that a multiscale controller design should be attempted. The experimental data was then subjected to multi-resolution decomposition and was indeed found to be highly multiscale [23]. After some debate, it was suggested to refocus the effort from model-based robust controller improvement and traditional constrained MPC to empirical multiresolution controller development. The initial step was to fit a multiresolution nonlinear, but linear in parameters, temporal model to the input/output data, with the subsequent steps involving the self-tuning GPC-type controller development, as shown in Figure 3. After a number of prolonged experiments, the resulting controller was outfitted with rate constraints and tuned to work on the test rig, showing performance noticeably superior to that of the other techniques employed. Further on, since the underlying process dynamics was exhibited by the intrinsically spatially distributed processes, a spatiotemporal multiresolution control component was developed for the riser dynamics and combined with the temporal topology. The latter part was implemented and tested, producing reasonable overall performance, but it could not be fully utilized due to the computational real-time controller limitations. Subsequently, the results presented in this paper were acknowledged by ALSTOM as making a real breakthrough in the project. The findings and their implications in the broad context imply that if a robust linear controller does not work well on a system, the system is likely rather complex, and the nonlinear multiresolution modeling in combination with linear-type control structures under constraints could offer effective configurations for high performance system control.

The future theoretical effort in the temporal implementation in Section 3 should involve developing the proof of the simultaneous error convergence of the coupled identifier/controller system under control rate constraints—a challenging analytical task. Future research should also address spatiotemporal controller development and implementation in more generality than that presented in Section 4. In the present work, the latter controller had limited efficacy due to the low sampling/computation rate of the test rig control processor, but the approach presented stands ready for further theoretical advancement and applications involving more advanced hardware.

Due to the combination of the coarser temporal and the finer spatiotemporal control in the overall control configuration, the results presented have a broad appeal in other applications involving complex multiscale spatiotemporal dynamics, such as, for example, robotic electrosurgery [41]. In this application, the motion of the cutting probe is strongly coupled to the spatiotemporal electrosurgical impact of the latter on the target tissues, and the near-field probe-tissue interaction process is best described by a complex time-fractional PDE [42]. These features make the technique proposed a good match for this application.

## 6. Conclusions

In this paper, the following results have been presented:

- closing the gap between the actual system output data and that predicted by the first-principles model of the complex chemical looping process through the empirically identified wavelet multiresolution analysis model initially trained on-line to estimate the nonlinear dynamic characteristics;
- embedding the multiresolution model into the generalized predictive control structure to obtain self-tuning control strategy for stable tracking of a chemical looping process with complex process dynamics under actuator rate constraints;
- showing boundedness of the adaptation gains for identification and control laws using the Lyapunov function theorems, and providing a guidance for attainment of asymptotic stability of the closed-loop system through the choice of these adaptation gains;
- using a spatiotemporal wavelet decomposition of the impulse response of the chemical looping process riser for designing a deadbeat predictive controller for further enhancement of the closed loop performance to account for fast system dynamics.
- experimentally confirming the effectiveness of the proposed controller design methods though their implementation on the novel single loop chemical looping cold flow testbed with complex dynamics,

Limitations of the present study lie in the insufficient spatiotemporal modeling and controller design for the riser and the inability to fully utilize the designed spatiotemporal controller for it because of the insufficient real-time performance of the control processor and the data acquisition system. Future efforts should focus on the advancement of the spatiotemporal part of the design, the overall controller robustness evaluation and enhancement, the development of the rigorous convergence proofs for the coupled identification/control laws under control rate constraints for the temporal and the entire spatiotemporal control topologies, and the implementation of the controller using advanced processors. The proposed techniques is planned to be applied by the authors to other areas, such as robotic electrosurgery.
